# A biplot correlation range for group-wise metabolite selection in mass spectrometry

**DOI:** 10.1186/s13040-019-0191-2

**Published:** 2019-02-04

**Authors:** Youngja H Park, Taewoon Kong, James R. Roede, Dean P. Jones, Kichun Lee

**Affiliations:** 1 College of Pharmacy, Korea University, Sejong, 30019 South Korea; 2 Industrial and Systems Engineering, Georgia Institute of Technology, Atlanta, GA 30332 USA; 3 Skaggs School of Pharmacy and Pharmaceutical Sciences, University of Colorado, Denver, CO 80045 USA; 4 Clinical Biomarkers Laboratory, Division of Pulmonary, Allergy and Critical Care Medicine, Atlanta, GA 30322 USA; 5 Department of Medicine, Emory University, Atlanta, GA 30322 USA; 6 Department of Industrial Engineering, Hanyang University, Seoul, 04763 South Korea

**Keywords:** Feature selection, Biplot correlation, Metabolomics

## Abstract

**Background:**

Analytic methods are available to acquire extensive metabolic information in a cost-effective manner for personalized medicine, yet disease risk and diagnosis mostly rely upon individual biomarkers based on statistical principles of false discovery rate and correlation. Due to functional redundancies and multiple layers of regulation in complex biologic systems, individual biomarkers, while useful, are inherently limited in disease characterization. Data reduction and discriminant analysis tools such as principal component analysis (PCA), partial least squares (PLS), or orthogonal PLS (O-PLS) provide approaches to separate the metabolic phenotypes, but do not offer a statistical basis for selection of group-wise metabolites as contributors to metabolic phenotypes.

**Methods:**

We present a dimensionality-reduction based approach termed ‘biplot correlation range (BCR)’ that uses biplot correlation analysis with direct orthogonal signal correction and PLS to provide the group-wise selection of metabolic markers contributing to metabolic phenotypes.

**Results:**

Using a simulated multiple-layer system that often arises in complex biologic systems, we show the feasibility and superiority of the proposed approach in comparison of existing approaches based on false discovery rate and correlation. To demonstrate the proposed method in a real-life dataset, we used LC-MS based metabolomics to determine spectrum of metabolites present in liver mitochondria from wild-type (WT) mice and thioredoxin-2 transgenic (TG) mice. We select discriminatory variables in terms of increased score in the direction of class identity using BCR. The results show that BCR provides means to identify metabolites contributing to class separation in a manner that a statistical method by false discovery rate or statistical total correlation spectroscopy can hardly find in complex data analysis for predictive health and personalized medicine.

**Electronic supplementary material:**

The online version of this article (10.1186/s13040-019-0191-2) contains supplementary material, which is available to authorized users.

## Introduction

Contemporary analytic methods, such as liquid chromatography-mass spectrometry (LC-MS) [1, 2], gas chromatography-mass spectrometry (GC-MS) [3, 4], and proton nuclear magnetic resonance (1H NMR) spectroscopy [5, 6], provide information-rich data sets that can be of substantial value in biomedical research and, in principle, can be developed with bioinformatics procedures for routine healthcare [7–9]. Challenges in clinical use exist at two levels, reliable extraction of metabolic features from spectroscopic data and reliable identification of metabolic features associated with health characteristics. Substantial progress has been made in data extraction, with several high-quality routines available. For instance, recent introduction of adaptive processing by apLCMS [10] provides a systematic approach to reduce noise and extract relative quantification of > 7000 metabolic features in 50 aliquots of human plasma in 20 min (2); current improvements in data processing have demonstrated that > 12,000 metabolic features can be extracted [11]. This high volume of information, which is inherently multivariate, presents challenges to reliable use in health prediction and disease management.

Statistical methods based upon the principles of false discovery rate (FDR) are available to correct for large numbers of comparisons in multiple hypothesis testing of metabolomics data [12]. These methods are useful to identify potential biomarkers associated with disease or disease risk while controlling the expected proportion of incorrectly rejected null hypotheses (type-I error). This approach is effective because it yields single biomarker candidates that can be rigorously tested and directly used in health research and clinical practice.

Individual biomarkers, however, can be of limited value in practical use. For instance, biomarkers with a relatively good specificity (e.g., 0.9) and sensitivity (e.g., 0.9) still result in large numbers of misclassifications, i.e., one diagnosis in ten will be wrong and one in ten will be missed. While several factors can contribute, a central limitation is that statistical procedures examining individual variables do not consider how variables interact and combine. In complex pathobiology, individuals with the same genetic mutation have different disease phenotypes, e.g., some patients with a sickle cell disease mutation have hemolytic crises while others with the same mutation have painful crises with bony infarcts, acute chest syndrome or only mild anemia [13]. At the molecular level, functional redundancies and multiple interacting levels of regulation within network structures result in second-order and higher order interactions that allow the same pathway to respond differently among individuals. Additionally, metabolic responses can be conditional because of genetic and epigenetic differences, as well as differences in diet, environment or health behaviors. For instance, decreased plasma cystine in response to zinc supplementation may not only be due to zinc-dependent effects on cystine uptake and conversion to glutathione by tissues [14, 15], but also upon intestinal absorption, renal loss, rates of transcription of relevant regulatory systems and past exposures that alter epigenetic regulation [16, 17]. Such complexity means that individual biomarkers can rarely, if ever, be universally useful. Consequently, statistical approaches equivalent to FDR, when conducting multiple comparisons, are needed to identify metabolites important in group-wise (e.g., metabolic pathway and network) behavior, thereby providing rigorous bases to include metabolic interactions within complex metabolic datasets for improved disease classification and health prediction.

In this study, we propose a general dimensionality-reduction based approach for potential biomarker selection in spectroscopic data, which we term ‘biplot correlation range’ (BCR). The approach uses loading vectors from principal component analysis (PCA), partial least squares (PLS, also called projection to latent structures), and orthogonal-signal-correction PLS (OPLS), to directly link to correlation analysis for group separation. The analysis determines a correlation range from scores for a group label on loading vectors rather than from individual correlations for each variable. The use of correlation range to describe how variables combine to form observable and discriminatory patterns is derived from established data reduction and multivariate techniques, (i.e., PCA, PLS, and OPLS), and methods to discover new variables describing otherwise hidden, lower-dimensional structure. Extracted representations are transformed into new data (score vectors) using a relatively small number of newly selected variables (loading vectors comprised of the original variable contributions). These new variables have improved power to discriminate samples linked to phenotype, such as pathological characteristic (Y as response variable). A specific advantage of this approach is that multiple components, none of which may be significantly associated with Y when evaluated individually by statistical tests such as FDR, can interact to discriminate Y using BCR.

In development of the proposed approach, we relied upon a commonly used graphical technique of matching score plots and loading plots, called as a biplot method [18]. Cloarec et al. used a two-step approach to facilitate biomarker detection in 1H NMR spectroscopy by graphically coupling a loading vector from OPLS and the correlation of each variable with response Y [19]. In the development of BCR, we similarly create a loading vector for each metabolite contributing to separation. A subsequent selection of metabolites with defined correlation interval (e.g., 95%) is used to determine metabolites related with defined classes. This allows individual metabolites contributing to separation to be visualized in respective loading plots, thereby providing a rigorously defined approach to identify metabolites contributing to group behavior. We explore whether BCR would determine a correlation range using scores and loadings in PCA, extending them to PLS and OPLS, and biomarkers for the purpose of discrimination analysis of mass spectral data from mitochondria isolated from wild-type (WT) mice and thioredoxin-2 (Trx2) TG mice. This study showed that BCR provides means to select metabolites contributing to class separation in a manner that can complement FDR in complex data analysis for predictive health and personalized medicine.

## Methods

In this section, we introduce the theoretical background of a biplot and its interpretation from a correlation viewpoint with regard to the development of biplot correlation statistics.

### Biplot

A biplot is constructed by using a dimensionality-reduction technique to obtain a low-dimensional approximation to a transformed version of a data matrix, **X** in size *n* × *p*, where n and p denote the number of samples (observations) and features (variables), respectively. The most popular dimensionality-reduction technique is singular value decomposition (SVD) which brings forth principal component analysis (PCA). Other techniques such as multidimensional scaling and partial least squares (PLS, also called projection to latent structures) are also available [20–22]. They, however, share the same spirit with SVD in a sense that the low-dimensional approximation of **X** often unravels hidden structures in **X** by maintaining inter-sample distances as much and capturing as much variation of **X** as possible.

For *n* centered *p* × 1 observations **x** ***=*** [x_1_⋯x_*p*_]^T^ and its corresponding *n* × 1 response vector **Y**, data matrix **X** consists of **x**_i_, i = 1, … , *n* (all up to *n* samples): **X =** [**x**_1_**⋯x**_*n*_]^T^. We find loading vectors ***a***_j_ of size *p* × 1, j = 1, … , *p*, and their associated score vectors **t**_j_ of size *n* × 1. Using SVD, we exactly obtain loading vector ***a***_j_ by an eigenvector of the sample covariance matrix and score vector **t**_j_ by

$$ {\mathbf{t}}_{\mathrm{j}}=\mathbf{X}{\mathbf{a}}_{\mathrm{j}}={\left[{\mathbf{a}}_{\mathrm{j}}^{\mathrm{T}}{\mathbf{x}}_1\cdots {\mathbf{a}}_{\mathrm{j}}^{\mathrm{T}}{\mathbf{x}}_n\right]}^{\mathrm{T}}. $$

Score vector **t**_j_ corresponding to loading vector **a**_j_ represents new coordinates of *n* data on the axis of **a**_j_. The *m*th component [**a**_j_]_m_ describes the amount of contribution of the original (before-transformation) *m*th variable to the construction of new axis **a**_j_.

Simply speaking, a larger [**a**_j_]_m_ value is associated with more weight for the *m*th variable in new axis **a**_j_. In practical use, before the application of dimensionality reduction, one could apply unit variance scaling for each column in **X** to provide all variables an equal weight. This scaling step, optional, depends on the domain characteristic of the data. For example, many biologic processes are determined by high abundance components, and because of this, variance scaling can sometimes result in loss of useful information by decreasing the contribution of more relevant, high-abundance variables and increasing contribution of non-relevant, low abundance variables.

**Fig. 1:**
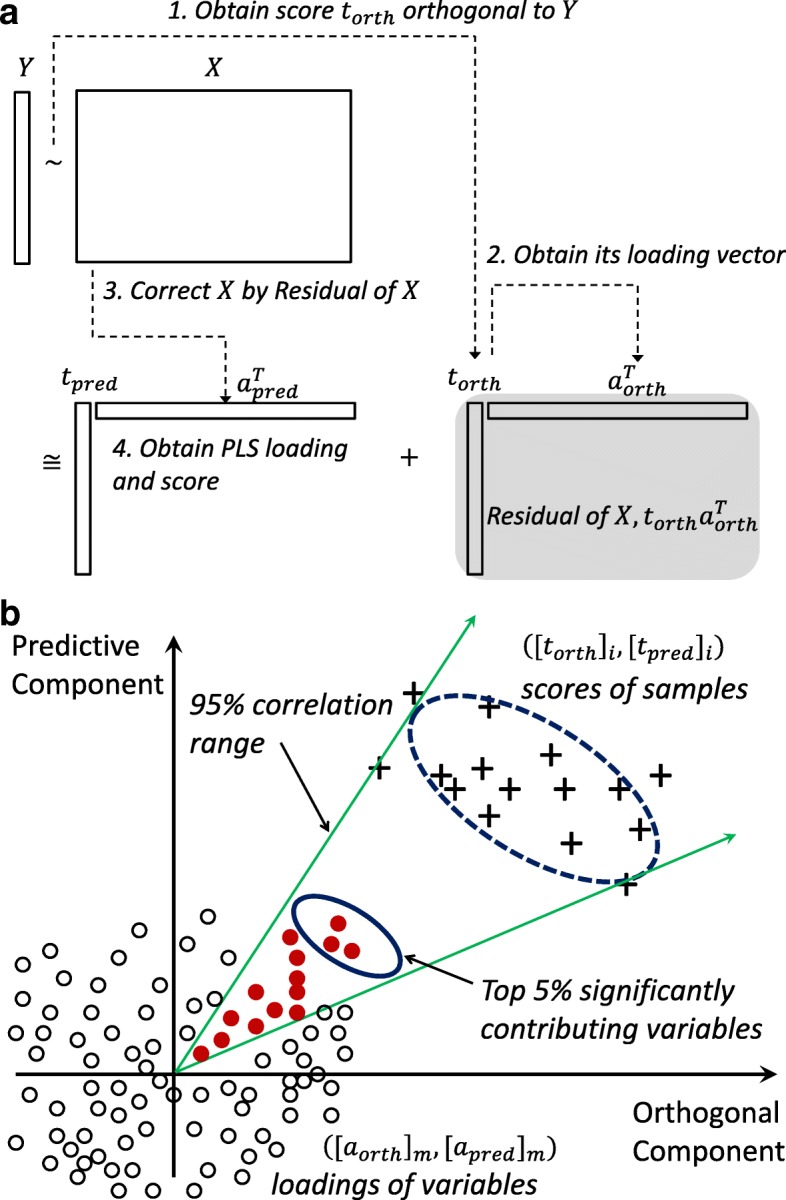
**a** The combination of a principal component score plot and a principal component loading plot, called a biplot, is used to illustrate the concept of biplot correlation range. Based on the “+” score plot values and the associated ellipse (broken line), a 95% confidence interval is calculated for projection onto the corresponding loading plot (small circles). Filled red circles contribute to scores within the 95% correlation range, while the thick-lined ellipse represents the top 5% of features contributing to the “+” class. **b** The flow of the decomposition of input data X

We order loading vectors, ***a***_1_**,** …**,** ***a***_*p*_**,** according to their associated eigenvalues and *p* score vectors, **t**_1_**, …, t**_*p*_ will follow accordingly. Then we rewrite the data matrix X as $\mathbf{X}={\sum}_{\mathrm{j}=1}^p{\mathbf{t}}_{\mathrm{j}}{\mathbf{a}}_{\mathrm{j}}^{\mathrm{T}}.$ A biplot is formed by the first two dominant terms from two scatterplots of ([**t**_1_]_i_, [**t**_2_]_i_) for i = 1, …, *n*, and ([**a**_1_]_m_, [**a**_2_]_m_), denoted by ${\overrightarrow{\mathbf{a}}}_{\mathrm{m}}$, for m = 1, …, *p* that share a common set of axes. In essence, excluding the constant term, the sample covariance matrix approximates to ${\mathbf{X}}^{\mathrm{T}}\mathbf{X}\cong {\mathbf{a}}_1{\mathbf{t}}_1^{\mathrm{T}}{\mathbf{t}}_1{\mathbf{a}}_1^{\mathrm{T}}+\mathbf{\cdots}+{\mathbf{a}}_{\mathrm{k}}{\mathbf{t}}_{\mathrm{k}}^{\mathrm{T}}{\mathbf{t}}_{\mathrm{k}}{\mathbf{a}}_{\mathrm{k}}^{\mathrm{T}}$**.** Fig. 1 (a) shows an exemplary biplot that has the simplified combination of a principal component score plot by ‘+’ markers and a principal component loading plot by ‘o’ markers.

### Biplot correlation range

We present an interpretation of the direction and magnitude in a biplot in terms of correlation among variables and relate it to a procedure for biplot based discrimination analysis. Using the approximation of the sample covariance matrix in the construction of a biplot, the sample covariance between the *q*th and *r*th variables, $\widehat{\operatorname{cov}}\ \left({\mathrm{x}}_{\mathrm{q}},{\mathrm{x}}_{\mathrm{r}}\right)$, is given by

$$ \widehat{\operatorname{cov}}\ \left({\mathrm{x}}_{\mathrm{q}},{\mathrm{x}}_{\mathrm{r}}\right)={\left[{\mathbf{X}}^{\mathrm{T}}\mathbf{X}\right]}_{\mathrm{q},\mathrm{r}}\cong {\left[{\mathbf{a}}_1{\mathbf{t}}_1^{\mathrm{T}}{\mathbf{t}}_1{\mathbf{a}}_1^{\mathrm{T}}\right]}_{\mathrm{q},\mathrm{r}}+{\left[{\mathbf{a}}_2{\mathbf{t}}_2^{\mathrm{T}}{\mathbf{t}}_2{\mathbf{a}}_2^{\mathrm{T}}\right]}_{\mathrm{q},\mathrm{r}}={\left[{\mathbf{a}}_1\right]}_{\mathrm{q}}{\mathbf{t}}_1^{\mathrm{T}}{\mathbf{t}}_1{\left[{\mathbf{a}}_1\right]}_{\mathrm{r}}+{\left[{\mathbf{a}}_2\right]}_{\mathrm{q}}{\mathbf{t}}_2^{\mathrm{T}}{\mathbf{t}}_2{\left[{\mathbf{a}}_2\right]}_{\mathrm{r}}=\left\langle {\overrightarrow{\mathbf{a}}}_{\mathrm{q}},{\overrightarrow{\mathbf{a}}}_{\mathrm{r}}\right\rangle $$

where 〈∙, ∙〉 represents an inner product with a weight vector $\left[{\mathbf{t}}_1^{\mathrm{T}}{\mathbf{t}}_1\ {\mathbf{t}}_2^{\mathrm{T}}{\mathbf{t}}_2\right].$It implies that the inner product between ${\overrightarrow{\mathbf{a}}}_{\mathrm{q}}$and ${\overrightarrow{\mathbf{a}}}_{\mathrm{r}}$ corresponds to a covariance measure between the two variables. Observe that ${\overrightarrow{\mathbf{a}}}_{\mathrm{q}}$ and ${\overrightarrow{\mathbf{a}}}_{\mathrm{r}}$ are shown as two loading vectors in a biplot. Then, given loading vector ${\overrightarrow{\mathbf{a}}}_{\mathrm{q}}=\left({\left[{\mathbf{a}}_1\right]}_{\mathrm{q}},{\left[{\mathbf{a}}_2\right]}_{\mathrm{q}}\right)$, it is straightforward that the direction of ${\overrightarrow{\mathbf{a}}}_{\mathrm{r}}$ should be the same as that of ${\overrightarrow{\mathbf{a}}}_{\mathrm{q}}$ to maximize

$$ {\left[{\mathbf{a}}_1\right]}_{\mathrm{q}}{\mathbf{t}}_1^{\mathrm{T}}{\mathbf{t}}_1{\left[{\mathbf{a}}_1\right]}_{\mathrm{r}}+{\left[{\mathbf{a}}_2\right]}_{\mathrm{q}}{\mathbf{t}}_2^{\mathrm{T}}{\mathbf{t}}_2{\left[{\mathbf{a}}_2\right]}_{\mathrm{r}}=\left\Vert {\overrightarrow{\mathbf{a}}}_{\mathrm{q}}\right\Vert\ \left\Vert {\overrightarrow{\mathbf{a}}}_{\mathrm{r}}\right\Vert \cos \theta \le \left\Vert {\overrightarrow{\mathbf{a}}}_{\mathrm{q}}\right\Vert\ \left\Vert {\overrightarrow{\mathbf{a}}}_{\mathrm{r}}\right\Vert, $$

where θ is the angle between the two vectors and the equality holds true for θ = 0. In specific, the cosine of the angle between ${\overrightarrow{\mathbf{a}}}_{\mathrm{q}}$ and ${\overrightarrow{\mathbf{a}}}_{\mathrm{r}}$ is related to a correlation measure,

$$ \widehat{\mathrm{corr}}\ \left({\mathrm{x}}_{\mathrm{q}},{\mathrm{x}}_{\mathrm{r}}\right)=\frac{\widehat{\operatorname{cov}}\ \left({\mathrm{x}}_{\mathrm{q}},{\mathrm{x}}_{\mathrm{r}}\right)}{\sqrt{\widehat{\operatorname{cov}}\ \left({\mathrm{x}}_{\mathrm{q}},{\mathrm{x}}_{\mathrm{q}}\right)\ \widehat{\operatorname{cov}}\ \left({\mathrm{x}}_{\mathrm{r}},{\mathrm{x}}_{\mathrm{r}}\right)}}\cong \frac{\left\langle {\overrightarrow{\mathbf{a}}}_{\mathrm{q}},{\overrightarrow{\mathbf{a}}}_{\mathrm{r}}\right\rangle }{\sqrt{\left\langle {\overrightarrow{\mathbf{a}}}_{\mathrm{q}},{\overrightarrow{\mathbf{a}}}_{\mathrm{q}}\right\rangle\ \left\langle {\overrightarrow{\mathbf{a}}}_{\mathrm{r}},{\overrightarrow{\mathbf{a}}}_{\mathrm{r}}\right\rangle }}=\cos \theta . $$

Overall, the direction of ${\overrightarrow{\mathbf{a}}}_{\mathrm{j}}$ for the *j*th variable linking to correlation of variables is the direction to which the variable contributes in increasing scores on new axes **a**_1_ and **a**_2_. Similarly, the magnitude of ${\overrightarrow{\mathbf{a}}}_{\mathrm{j}}$ is associated with the variable’s contribution in magnitude of the score increasing, linking to covariance of variables. For the sake of convenience, ${\overrightarrow{\mathbf{a}}}_{\mathrm{j}}$ is used as the contributing direction of the *j*th variable.

When dimensionality-reduction techniques such as PLS and OPLS other than SVD approximate data matrix **X** in a similar fashion, the BCR approach is applied similarly using their loading vectors. Among numerous dimensionality-reduction techniques implemented and tested, we choose the combination of direct signal correction and PLS to obtain components able to separate response vector **Y** with interpretability [23]. The flow of the decomposition is shown in Fig. 1 (b). We extract a signal score vector, **t**_(*ortho*)_, orthogonal to response vector **Y** with maximum variance in data matrix **X**. In specific, for $\widehat{\mathbf{Y}}$, the projection of **Y** on the column space of **X**, we numerically obtain the eigenvector with the largest eigenvalue, set to be **t**_(*ortho*)_, in a subspace of **X** orthogonal to $\widehat{\mathbf{Y}}$, $\left(\mathbf{I}-\widehat{\mathbf{Y}}{\left({\widehat{\mathbf{Y}}}^T\widehat{\mathbf{Y}}\right)}^{-\mathbf{1}}{\widehat{\mathbf{Y}}}^{\mathrm{T}}\right)\mathbf{X},$and then compute its loading vector ${\mathbf{a}}_{(ortho)}={\mathbf{X}}^{\mathrm{T}}{\mathbf{t}}_{(ortho)}{\left({\mathbf{t}}_{(ortho)}^{\mathrm{T}}{\mathbf{t}}_{(ortho)}\right)}^{-\mathbf{1}}$. We consider ${\mathbf{t}}_{(ortho)}{\mathbf{a}}_{(ortho)}^{\mathrm{T}}$ as a residual in **X** in the task of explaining **Y**. Then we perform PLS with input data as $\mathbf{X}-{\mathbf{t}}_{(ortho)}{\mathbf{a}}_{(ortho)}^{\mathrm{T}}$, corrected by the **Y**-orthogonal signal in **X**, and output data **Y**, obtaining its first score vector, **t**_(*pred*)_**,** and loading vector, **a**_(*pred*)_. We note that the use of direct signal correction in **X** by the first orthogonal component beforehand helps the first component of PLS to effectively capture **Y-**separating patterns in **X** and produces interpretability when using the two components. Finally, we approximate data matrix **X** into one orthogonal component and another predictive component as follows:

$$ \mathbf{X}\cong {\mathbf{t}}_{(pred)}{\mathbf{a}}_{(pred)}^{\mathrm{T}}+{\mathbf{t}}_{(ortho)}{\mathbf{a}}_{(ortho)}^{\mathrm{T}}. $$

Score and loading vectors in the above decomposition retain the same interpretation as in PCA. We notice that the decomposition without an orthogonal component is equivalent to PLS and that numerous orthogonal and PLS components are possible in addition to various feature scaling methods. Typically, some kind of cross validation in combination with the domain characteristic of features is used to optimize the separability of the whole procedure.

Then, using the properties of loading vectors as discussed above, variables contributing significantly to a certain group label can be identified as in Fig. 1. First, we construct a biplot as described before and collect the scores of samples belonging to a certain group label. Often score vectors are scaled appropriately so that they may be placed outside loading vectors for the sake of clarity. By default, we multiplied the scores by 0.001. As the next step shows, since we consider loading vectors among themselves using their directions and magnitudes, such scaling does not matter. Then we use a 95% confidence interval fitting the scores belonging to a certain group with a multivariate normal distribution, forming a correlation range based on the angle interpretation in biplots. In Fig. 1, it is illustrated by the ‘+’ markers representing scores of samples belonging to a group and the ellipsoid with a broken line representing a 95% confidence interval for those scores. Next, variable *j* corresponding to direction ${\overrightarrow{\mathbf{a}}}_{\mathrm{j}}$ that contributes to increasing scores within the 95% confidence interval range (edge bordered by diagonal green lines in Fig. 1), are collected. The filled red circles in Fig. 1 illustrate collected variables. The two (green) diagonal solid lines that border on the 95% confidence interval represent a biplot correlation range (BCR). We notice that the correlation range is invariant to the scaling of score vectors in consideration of the diagonal lines from the zero. Finally, the top 5% (τ) of variables in magnitude of contributing direction ${\overrightarrow{\mathbf{a}}}_{\mathrm{j}}$ are selected. Note that the stringency can be increased by use of top 1% or 0.5% of such variables. These features, illustrated as filled circles in the solid ellipsoid, greatly contribute to the group label in a covariance (magnitude) sense. We perform the above procedures with scores of each group label, generating selected features per group label, as in Figs. 3(a) and 5(a), and finally obtaining the union of them. Since the selected features are treated equally as long as they are within the top 5% criterion, post-analysis such as ordering them by correlation, covariance, *p*-values, or variable importance projection (VIP) values will be possible [24]. By default, we filter out variables of which individual regression performance measures with response **Y** are weak. Practically, we test if the p-value of a logistic regression model with response **Y** and the raw values of each individual variable x_i_ is greater than 0.10, without controlling familywise error rate or false discovery rate, to deem features of weak separability. Depending on the nature of variables and the problem domain, one could adopt other performance measures and tests such as Pearson correlation, Spearman’s rank correlation, *p* values of linear regression, and classification accuracy of logistic regression. This step eliminates unnecessary noisy features that act as contributing features in a collective sense from the previous step. We repeat the above steps for each group label, and the outcomes of these steps are lists of greatly contributing features for each group label.

This BCR approach is based on the graphical use of a biplot in that scores and loadings are used collectively. BCR, however, enables discrimination analysis and a feature selection procedure using the interpretation of loading vectors while the biplot approach provides only a graphical presentation of scores and loadings. We note that BCR relies upon the approximation of data matrix $\mathbf{X}\cong {\mathbf{t}}_1{\mathbf{a}}_1^{\mathrm{T}}+{\mathbf{t}}_2{\mathbf{a}}_2^{\mathrm{T}}$ using orthogonal and predictive components and the statistical properties of loading vector ${\overrightarrow{\mathbf{a}}}_{\mathrm{j}}$ and score vector ${\overrightarrow{\mathbf{t}}}_{\mathrm{j}}$ and it provides a structured approach to select features collectively significant.

## Results

To test the BCR approach, we performed a simulation study and compared it with some existing methods. Then, we applied it to a real-life example.

### Simulation and comparisons

**Fig. 2:**
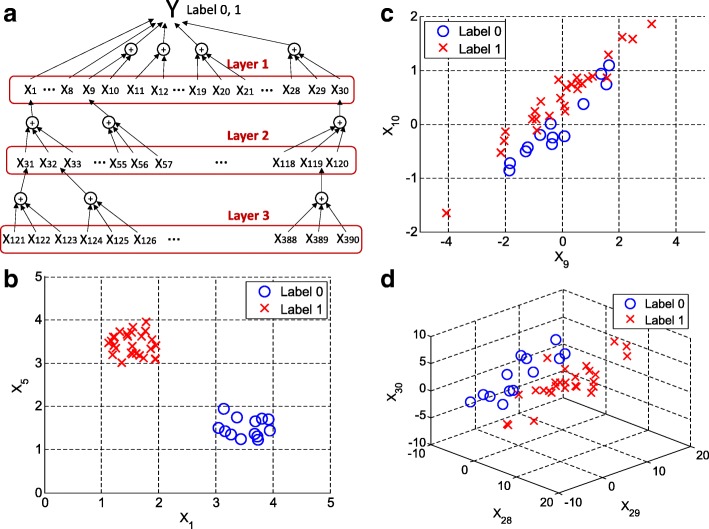
**a** A data model was created for 200 samples, each with 1000 measured variables. The model has three layers of interaction of variables x that contribute to the response Y. Layer 1 contains 30 variables, including eight strong variables (x_1_ to x_8_) and other group-wise variables (x_9_ to x_30_). Each of the variables in Layer 1 is determined by three variables in Layer 2, and each in Layer 2 by three variables in Layer 3. The remaining 610 variables are randomly assigned. **b** Two strong variables, x_1_ and x_5_, individually separate the two labels of response Y. **c** Two strong variables, x_9_ and x_10_, jointly separate the two labels of response Y. (d) Three variables x_9_, x_10_, and x_11_ in Layer 1, jointly separate the two labels of response Y

We first generated a data matrix **X** (200 × 1000) and a response vector **Y** (200 × 1), comprising 200 samples and 1000 variables. Each element of **Y** was a Bernoulli random variable with a success probability of 0.4, so 16 elements of **Y** were set to 1 on average, while the remaining 24 elements were set to 0. To generate 1000 variables, we used a three-layer network structure shown in Fig. 2.

In the first layer, the first 30 variables were generated to have high separation in **Y**: for *p* = 1, …, 4,

$$ {\mathrm{x}}_{\mathrm{p}}\sim \mathrm{U}\left(0,1\right)+0.8-2\mathrm{Y}, $$

for *p* = 5, …, 8,

$$ {\mathrm{x}}_{\mathrm{p}}\sim \mathrm{U}\left(0,1\right)-1.2-2\mathrm{Y}, $$

where U(a, b) is a random variable from a uniform distribution of range a and b. It means variables from x_1_ to x_4_ individually had a high positive correlation with **Y** label 0, whereas variables x_5_ to x_8_ highly correlated with **Y** label 1. In specific, the correlation between each of variables from x_1_ to x_4_ and **Y** was 0.9576 ± 0.0075 (average ± standard deviation) throughout the simulation, and that between each of variables x_5_ to x_8_ and **Y** was 0.9577 ± 0.0075. Figure 2(b) shows plots of realizations of x_1_ and x_5_ and the aforementioned pattern that clearly and individually separate **Y**. The first eight variables in the first layer represent strong individual variables that should be used to identify pathological conditions. For *p* = 9, 12, …, 18,

$$ {\left[{\mathrm{x}}_{\mathrm{p}}\ {\mathrm{x}}_{\mathrm{p}+1}\right]}^{\boldsymbol{T}}\sim \mathrm{N}\left(0+\mathrm{Y}{\left[0\ 0.5\right]}^T,\mathrm{A}\right), $$

and for *p* = 19, 22, 25, 28,

$$ {\left[{\mathrm{x}}_{\mathrm{p}}\ {\mathrm{x}}_{\mathrm{p}+1}\ {\mathrm{x}}_{\mathrm{p}+2}\right]}^{\boldsymbol{T}}\sim \mathrm{N}\left(0+\mathrm{Y}{\left[1\ 2\ 3\right]}^T,\mathrm{B}\right), $$

where $$ \mathrm{A}=\left[\begin{array}{cc}1& 0.4\\ {}0.4& 0.4\end{array}\right], $$ $$ \mathrm{B}=\left[\begin{array}{ccc}12& 10& 8\\ {}10& 12& 10\\ {}8& 10& 12\end{array}\right], $$and N(μ, ∑) represents a multivariate normal distribution with mean μ and covariance ∑. Figure 2(c) shows plots of realizations of x_9_ and x_10_ that collectively separate **Y**. Figure 2(d) also illustrates that realizations of x_28_, x_29_ and x_30_, generated as above, clearly and jointly discriminate **Y.** The variables x_i_, i = 9, …, 30, in the first layer represent strong group-wise variables that clearly discriminate pathological conditions. The next 90 variables from x_31_ to x_120_ in the second layer and the next 270 variables from x_121_ to x_390_ in the third layer were generated so that the variables contribute to the overall response **Y** in a composite and aggregate manner. For instance, x_1_ in the first layer is clearly separable by the combination of x_31_, x_32_ and x_33_, in the second layer. In specific, the generation of the three variables is based on the value of x_1_ so that the sum of the three will be close to x_1_ as follows: given x_1_, we independently generate u_1_, u_2_ and u_3_ from U(0,1) and ϵ from, $\mathrm{N}\left(0,\frac{x_1}{10}\right)$. Then, we set

$$ {\mathrm{x}}_{31}=\frac{u_1}{u_1+{u}_2+{u}_3}\ \left({\mathrm{x}}_1+\upepsilon \right), $$

$$ {\mathrm{x}}_{32}=\frac{u_2}{u_1+{u}_2+{u}_3}\ \left({\mathrm{x}}_1+\upepsilon \right), $$

$$ {\mathrm{x}}_{33}=\frac{u_3}{u_1+{u}_2+{u}_3}\ \left({\mathrm{x}}_1+\upepsilon \right), $$

which brings x_31_ ***+*** x_32_ ***+*** *x*_33_ ***=*** x_1_ ***+*** ϵ. The correlation between the sum of the three and x_1_ was 0.9730 ± 0.0085 throughout the simulation, and the generation method was similarly applied to the according matches in Fig. 2 (a). The remaining 610 variables from x_391_ to x_1000_, comprising a noise layer, were randomly and independently generated from N(0, 1) to avoid a strong correlation with **Y** in order to simulate inherent noise. To better simulate the effect of randomness in noise, the *p*-value of a linear-regression fitting of **Y** with each x_*i*_ from the 610 variables was controlled so that p-value ≥*δ*_*i*_, where *δ*_*i*_ was set to 0, 0.03, 0.05, or 0.10. The condition, *δ*_*i*_=0, represents that the variables are completely random noise and some of them can be significant, and the condition, *δ*_*i*_=0.10, represents the generated variables are deemed insignificant by significance level 0.10. The simulated three-layer structure is an example of multiple layers of metabolic interactions and regulation in complex biological systems. For instance, a biological system for nutritional metabolomics reflects such a layered structure with linked transports [25]. To further simulate biological systems with fewer biomarkers, we also used the two-layer structure of layers 1 and 2 only, in which the remaining 880 variables were simulated noise, and the one-layer structure of only layer 1, in which the remaining 930 variables were simulated noise. To compare them with random-noise systems as a baseline performance, we also used a structure, denoted by noise layer structure, in which the total 1000 variables are simulated noise. The variables as simulated noise were generated as described above by varying *δ*_*i*_.

For comparison, we examined the ability of statistical total correlation spectroscopy coupled with OPLS (STOCSYO) [19] and false discovery rate (FDR) methods to detect the known variables from layers 1, 2, and 3. Statistical total correlation spectroscopy is an analysis method for aiding the identification of potential biomarkers in metabolomics studies by displaying the correlation among the intensities of the various peaks among the whole sample, and its combination with OPLS discriminant analysis, in particular, offers a powerful framework for selecting important variables [26, 27]. We used two versions of FDR using *p*-values from *t*-tests with two unpaired sets of x_*i*_ values when **Y**=0 and those when **Y**=1; one is a classic one (FDR1) by Benjamini and Hochberg [12] and the other one (FDR2), by Benjamini and Yekutieli, considers multiple testing under dependency [28]. We also mention that another version of FDR using p-values from logistic regression was tested but that no variables are found throughout the experiments. Thus, we dropped logistic regression based FDR in the following results. We chose STOCSYO as a representative method using correlation measures and FDR as using p-values. The BCR approach used the first orthogonal and the first predictive components from OPLS. The selection of important variables in STOCSYO was set as the cut-off value of a correlation coefficient corresponding to significance levels varying from 1 to 20% [19, 26, 29]. The top percentile (τ) in BCR and the q-value for FDR also varied from 1 to 20%. We note that the adopted levels for the methods are not strictly comparable metrics by themselves, yet we compare them in that they are used in practice to adjust the number of selected variables.

**Fig. 3:**
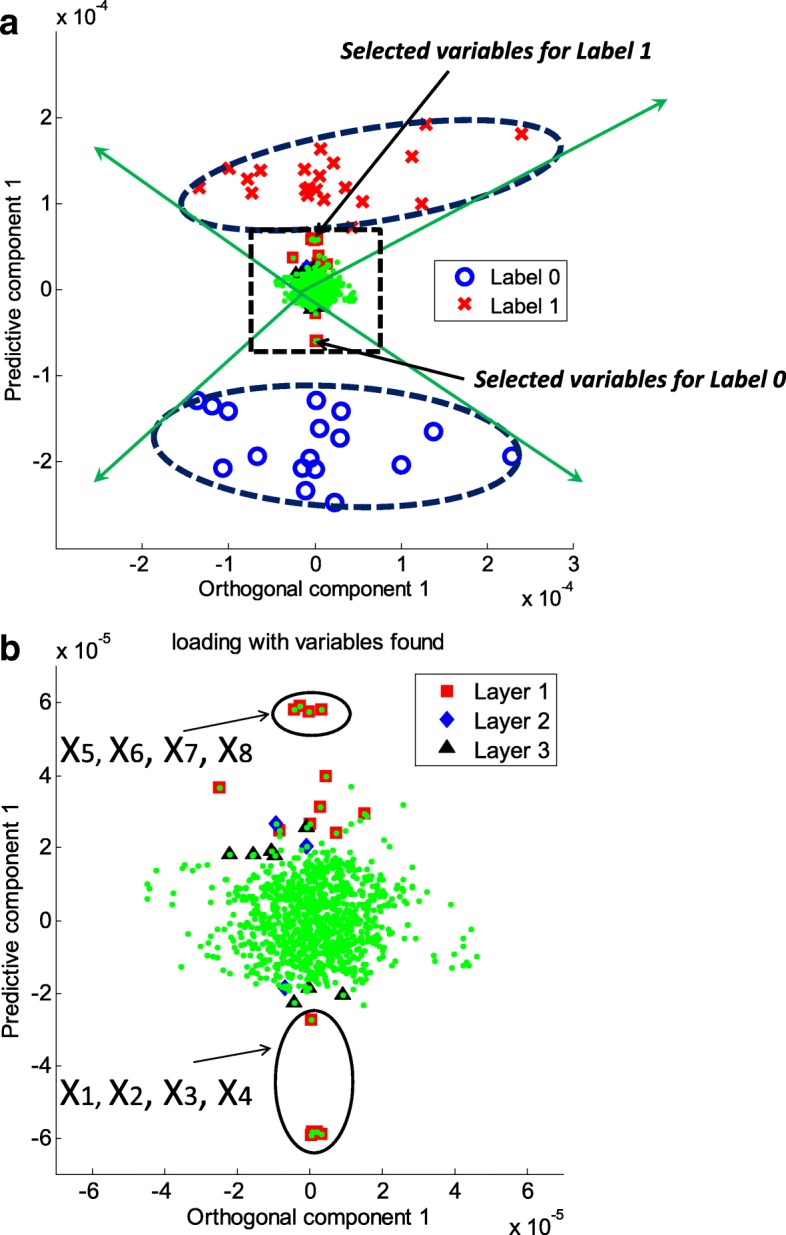
This figure illustrates feature selection using BCS for a simulated data set from the model in Fig. 2. **a** A biplot for the first orthogonal and predictive components is shown, clearing separating the two labels of response Y with a 95% correlation range for each label. **b** The corresponding loading plot, a zoomed-in version of the dotted-line rectangle in (**a**), is shown; the top 5% significantly contributing variables found from Layer 1, 2, and 3 are shown as red squares, blue diamonds, and black triangles, respectively. The eight strong variables (x_1_ to x_8_) in the plot are clearly distinguishable and positioned accordingly to the labels. For example, the position of x_1_ is aligned in the direction of label 0, consistent with the behavior of x_1_ in Fig. 2(b)

Figure 3 (a) and (b) show the scores and loadings, respectively, for the BCR method using a multiple-layer data set: in the loading plot, red squares, blue diamonds, and black triangles represent variables that are deemed to be greatly contributing from layers 1, 2, and 3, respectively. We note that the first eight variables (x_1_ to x_8_) were correctly found, and several additional variables from layers 2 and 3 were also detected. We notice that the loadings of the eight strong variables (x_1_ to x_8_) are greatly larger than those of others, for examples, layer 2 variables (x_9_ to x_30_) in the predictive component axis corresponding to loading vector **a**_(*pred*)_ as shown in Fig. 3 (b). It is understandable in view of that the loading vector in PLS is obtained as slope coefficients to predict **X** corrected by the **Y**-orthogonal signal, $\mathbf{X}-{\mathbf{t}}_{(ortho)}{\mathbf{a}}_{(ortho)}^{\mathrm{T}}$, by the score vector. We notice that the PLS score vector is calculated on the direction which maximizes the covariance between **X** and **Y**. It is worth mentioning that the first eight variables were positioned accordingly to the labels. The position of x_1_, for example, was aligned to the direction of label 0, in accordance with the behavior of x_1_ in Fig. 2 (b). This implies that an increase in x_1_ results in an increase in label 0.

We repeated this test 1000 times for each of the three kinds of layer structures. In each repetition for each method, we counted the numbers of distinguishing variables that were correctly found within the three layers. None of the noisy variables was selected by any of the methods for the three layers structures when *δ*_*i*_=0.10 while some noisy variables were selected for the other *δ*_*i*_ conditions. The averaged numbers found by the various methods in the three-layer, two-layer, one-layer, and noise-layer structure are presented in Tables 1, 2, 3, and 4, respectively. For the three-layer structure as shown in Table 1, the BCR method consistently found more variables in all the layers than STOCSYO and FDR. In layer 1, BCR outperformed STOCSYO and FDR for all levels and all noise conditions except level 0.01 with regard to the number of variables found. We also notice that the tested methods managed to find significant variables in layer 1, which is reasonable in that the methods are able to identify strong single variables. In layers 2 and 3, we observe the BCR method found more variables than the other methods. This result is understandable because BCR looks for a combination of variables rather than a single variable to separate labels, while STOCSYO emphasizes individual correlation-wise weights and FDR focuses on the effect of an outstanding single variable. In the noise layers, the BCR and STOC methods found more noise variables than the other methods for *δ*_*i*_ = 0, and the BCR only found noise variables for *δ*_*i*_ = 0.05. No noise variables were found for all the methods when *δ*_*i*_ = 0.10. This result implies that the BCR method tends to identify noise variables, possibly leading to false positives, when the randomness in noise increases. To validate the identified noise variables are false positives in discrimination analysis, we performed logistic regression for **Y** using the detected noise variables only. The *p*-values and classification accuracy are shown in Table 5 for the three-layer structure. Clearly, the logistic regression models are quite much significant with the *p*-values close to zero, and the classification performance increases as the number of the detected noise variable increases. It indicates that the detected noise variables are discriminative in the classification task. The p-values and classification accuracy for the two-layer, the one-layer, and the noise-layer structures in Additional files 1, 2 and 3: Tables S3–S5, respectively, show the similar result as in Table 5 for the detected noise variables.

**Table 1:** Average numbers of variables found in the simulation study for the three-layer structure

δ_*i*_	Level	Number of variables found for the three-layer structure															
		Layer 1				Layer 2				Layer 3				Noise Layer			
		BCS	FDR1	FDR2	STOC	BCS	FDR1	FDR2	STOC	BCS	FDR1	FDR2	STOC	BCS	FDR1	FDR2	STOC
*0*	*0.01*	16.4	**23.4**	21.1	10.0	**0.2**	0.0	0.0	0.0	**0.2**	0.0	0.0	0.0	**1.8**	0.1	0.0	0.0
	*0.03*	**24.2**	23.9	22.0	24.1	**2.8**	0.0	0.0	0.5	**5.1**	0.0	0.0	0.6	**18.2**	0.6	0.0	4.8
	*0.05*	**24.5**	23.8	22.0	24.2	**5.0**	0.1	0.0	1.8	**10.7**	0.0	0.0	3.1	**31.1**	0.8	0.0	16.4
	*0.07*	**24.8**	24.1	22.2	24.5	**5.6**	0.1	0.0	2.3	**12.7**	0.0	0.0	4.1	**39.8**	1.1	0.0	23.4
	*0.10*	**25.0**	24.3	22.2	24.6	**7.2**	0.2	0.0	3.3	**14.4**	0.1	0.0	5.0	**46.8**	1.7	0.0	28.3
	*0.15*	**25.2**	24.6	22.7	24.6	**7.0**	0.2	0.0	3.0	**14.8**	0.1	0.0	5.3	**47.5**	2.7	0.1	30.1
	*0.20*	**25.0**	24.5	22.7	24.5	**6.6**	0.2	0.0	3.0	**13.9**	0.2	0.0	4.6	**47.7**	4.3	0.1	29.9
*0.03*	*0.01*	10.0	**23.5**	21.1	16.9	**0.4**	0.0	0.0	0.0	**0.9**	0.0	0.0	0.0	**0.4**	0.0	0.0	0.0
	*0.03*	**24.6**	24.0	21.8	24.5	**3.5**	0.0	0.0	1.4	**7.3**	0.0	0.0	1.9	**10.5**	0.0	0.0	1.0
	*0.05*	**24.8**	23.9	22.2	24.4	**5.5**	0.1	0.0	2.5	**11.6**	0.0	0.0	3.8	**21.4**	0.0	0.0	6.1
	*0.07*	**24.8**	24.1	22.4	24.6	**6.0**	0.0	0.0	2.6	**12.4**	0.0	0.0	4.2	**30.0**	0.0	0.0	10.1
	*0.10*	**25.1**	24.4	22.6	24.7	**7.0**	0.1	0.0	3.0	**14.0**	0.1	0.0	4.5	**31.3**	0.0	0.0	11.5
	*0.15*	**25.1**	24.5	22.7	24.7	**6.6**	0.1	0.0	2.8	**14.5**	0.1	0.0	4.7	**34.5**	0.0	0.0	12.7
	*0.20*	**25.1**	24.4	22.8	24.7	**7.1**	0.1	0.0	3.3	**14.2**	0.1	0.0	4.8	**32.9**	0.0	0.0	11.8
*0.05*	*0.01*	10.0	**23.3**	21.0	16.6	**0.4**	0.0	0.0	0.0	**0.8**	0.0	0.0	0.0	**0.2**	0.0	0.0	0.0
	*0.03*	**24.5**	23.8	21.8	24.3	**4.3**	0.0	0.0	1.5	**8.2**	0.0	0.0	2.2	**6.0**	0.0	0.0	0.0
	*0.05*	**24.9**	24.2	22.2	24.6	**6.2**	0.0	0.0	2.7	**12.2**	0.0	0.0	4.3	**13.8**	0.0	0.0	0.0
	*0.07*	**25.0**	24.2	22.4	24.5	**6.4**	0.0	0.0	2.9	**13.5**	0.0	0.0	4.6	**19.6**	0.0	0.0	0.0
	*0.10*	**25.0**	24.2	22.4	24.3	**6.7**	0.1	0.0	2.9	**13.7**	0.0	0.0	4.9	**22.6**	0.0	0.0	0.0
	*0.15*	**25.1**	24.5	22.7	24.8	**7.1**	0.2	0.0	3.4	**14.6**	0.1	0.0	4.9	**24.2**	0.0	0.0	0.0
	*0.20*	**25.2**	24.5	22.8	24.7	**6.6**	0.2	0.0	3.0	**13.9**	0.1	0.0	4.6	**23.6**	0.0	0.0	0.0
*0.1*	*0.01*	16.8	**23.4**	21.2	10.0	**0.5**	0.0	0.0	0.0	**1.1**	0.0	0.0	0.0	0.0	0.0	0.0	0.0
	*0.03*	**24.5**	23.9	21.8	24.4	**4.8**	0.0	0.0	1.7	**9.3**	0.0	0.0	2.1	0.0	0.0	0.0	0.0
	*0.05*	**24.8**	24.0	22.0	24.4	**6.3**	0.1	0.0	2.8	**13.1**	0.0	0.0	4.4	0.0	0.0	0.0	0.0
	*0.07*	**25.0**	24.1	22.4	24.5	**6.2**	0.1	0.0	3.0	**13.0**	0.0	0.0	4.5	0.0	0.0	0.0	0.0
	*0.10*	**25.0**	24.3	22.3	24.4	**6.7**	0.1	0.0	3.0	**14.7**	0.0	0.0	5.0	0.0	0.0	0.0	0.0
	*0.15*	**25.1**	24.3	22.6	24.6	**6.8**	0.2	0.0	3.2	**13.5**	0.1	0.0	4.3	0.0	0.0	0.0	0.0
	*0.20*	**25.1**	24.5	22.9	24.7	**6.5**	0.1	0.0	2.8	**15.1**	0.1	0.0	5.0	0.0	0.0	0.0	0.0

**Table 2:** Average numbers of variables found in the simulation study for the two-layer structure

δ_*i*_	Level	Number of variables found for the two-layer structure											
		Layer 1				Layer 2				Noise Layer			
		BCS	FDR1	FDR2	STOC	BCS	FDR1	FDR2	STOC	BCS	FDR1	FDR2	STOC
*0*	*0.01*	16.9	23.2	21.0	10.0	0.2	0.0	0.0	0.0	2.9	0.2	0.0	0.0
	*0.03*	24.8	23.9	21.8	24.1	2.4	0.0	0.0	0.3	32.0	0.7	0.0	5.6
	*0.05*	25.0	24.0	22.1	24.7	5.3	0.0	0.0	1.8	61.7	1.1	0.0	22.9
	*0.07*	25.1	24.1	22.3	24.8	5.8	0.1	0.0	2.3	77.8	1.8	0.0	35.2
	*0.10*	25.2	24.2	22.4	25.0	7.1	0.2	0.0	2.8	87.2	2.4	0.1	44.0
	*0.15*	25.1	24.3	22.6	25.1	6.9	0.1	0.0	3.0	86.2	3.8	0.1	44.3
	*0.20*	25.3	24.6	22.8	25.2	7.0	0.3	0.0	2.9	85.8	6.2	0.1	44.2
*0.03*	*0.01*	17.6	23.4	21.3	10.0	0.7	0.0	0.0	0.0	1.6	0.0	0.0	0.0
	*0.03*	24.9	23.9	21.9	24.6	4.1	0.0	0.0	1.8	28.0	0.0	0.0	3.0
	*0.05*	25.1	24.1	22.2	24.9	6.2	0.0	0.0	2.9	49.6	0.0	0.0	12.0
	*0.07*	25.3	24.3	22.4	25.1	6.7	0.1	0.0	2.8	59.0	0.0	0.0	16.0
	*0.10*	25.3	24.2	22.5	25.0	7.1	0.1	0.0	3.2	62.2	0.0	0.0	17.7
	*0.15*	25.4	24.5	22.8	25.2	7.1	0.1	0.0	3.0	61.6	0.0	0.0	18.0
	*0.20*	25.4	24.5	22.9	25.1	6.7	0.2	0.0	2.7	62.5	0.0	0.0	18.6
*0.05*	*0.01*	17.5	23.1	21.1	10.0	1.1	0.0	0.0	0.0	1.2	0.0	0.0	0.0
	*0.03*	25.1	23.9	21.9	24.8	5.3	0.1	0.0	2.2	22.2	0.0	0.0	0.0
	*0.05*	25.1	24.1	22.3	24.9	6.6	0.1	0.0	3.0	36.9	0.0	0.0	0.0
	*0.07*	25.2	24.1	22.2	25.0	7.1	0.1	0.0	3.1	42.9	0.0	0.0	0.0
	*0.10*	25.2	24.3	22.5	25.1	7.2	0.0	0.0	2.9	44.8	0.0	0.0	0.0
	*0.15*	25.2	24.3	22.6	25.1	7.2	0.1	0.0	2.9	46.3	0.0	0.0	0.0
	*0.20*	25.2	24.4	22.9	25.1	6.8	0.2	0.0	2.9	44.6	0.0	0.0	0.0
*0.1*	*0.01*	17.8	23.3	21.1	10.0	1.5	0.0	0.0	0.0	0.0	0.0	0.0	0.0
	*0.03*	25.2	23.8	22.1	24.8	6.5	0.0	0.0	2.7	0.0	0.0	0.0	0.0
	*0.05*	25.2	23.9	22.0	25.1	7.0	0.0	0.0	3.1	0.0	0.0	0.0	0.0
	*0.07*	25.2	24.1	22.2	25.0	7.0	0.1	0.0	2.8	0.0	0.0	0.0	0.0
	*0.10*	25.2	24.2	22.7	25.1	6.6	0.1	0.0	2.8	0.0	0.0	0.0	0.0
	*0.15*	21.0	13.0	8.60	20.1	6.75	.087	.005	3.22	14.1	.043	.000	5.41
	*0.20*	21.0	13.5	8.72	20.0	6.52	.115	.003	2.98	14.4	.068	.000	5.27

**Table 3:** Average numbers of variables found in the simulation study for the one-layer structure

δ_*i*_	Level	Number of variables found for the one-layer structure							
		Layer 1				Noise Layer			
		BCS	FDR1	FDR2	STOC	BCS	FDR1	FDR2	STOC
*0*	*0.01*	17.1	23.3	21.4	10.0	2.9	0.2	0.0	0.0
	*0.03*	24.7	23.8	22.1	24.0	35.0	0.8	0.0	6.0
	*0.05*	25.2	24.1	22.5	24.6	68.9	1.2	0.0	24.8
	*0.07*	25.3	24.1	22.5	24.9	87.6	2.2	0.0	39.0
	*0.10*	25.4	24.4	22.6	25.1	96.2	2.7	0.1	45.9
	*0.15*	25.4	24.5	23.0	25.1	96.2	4.1	0.1	47.6
	*0.20*	25.4	24.7	23.0	25.2	96.6	6.9	0.1	48.4
*0.03*	*0.01*	17.6	23.3	21.3	10.0	2.4	0.0	0.0	0.0
	*0.03*	25.0	23.9	21.9	24.7	32.4	0.0	0.0	4.3
	*0.05*	25.3	24.1	22.0	25.1	56.3	0.0	0.0	14.1
	*0.07*	25.3	24.0	22.3	24.9	65.9	0.0	0.0	18.7
	*0.10*	25.3	24.3	22.5	25.1	67.8	0.0	0.0	20.0
	*0.15*	25.3	24.4	22.8	25.1	69.0	0.0	0.0	19.6
	*0.20*	25.5	24.6	23.1	25.2	71.2	0.0	0.0	20.4
*0.05*	*0.01*	17.7	23.1	21.1	10.0	2.2	0.0	0.0	0.0
	*0.03*	25.2	23.9	21.9	24.9	27.5	0.0	0.0	0.0
	*0.05*	25.3	24.0	22.0	25.0	43.1	0.0	0.0	0.0
	*0.07*	25.3	24.2	22.3	25.1	50.2	0.0	0.0	0.0
	*0.10*	25.4	24.4	22.7	25.1	50.6	0.0	0.0	0.0
	*0.15*	25.4	24.5	22.7	25.1	50.2	0.0	0.0	0.0
	*0.20*	25.4	24.5	22.9	25.1	51.3	0.0	0.0	0.0
*0.1*	*0.01*	17.8	23.2	21.0	10.0	0.0	0.0	0.0	0.0
	*0.03*	25.3	23.8	21.7	25.0	0.0	0.0	0.0	0.0
	*0.05*	25.2	24.0	22.1	25.1	0.0	0.0	0.0	0.0
	*0.07*	25.3	24.1	22.3	25.2	0.0	0.0	0.0	0.0
	*0.10*	25.3	24.3	22.5	25.2	0.0	0.0	0.0	0.0
	*0.15*	25.3	24.3	22.6	25.2	0.0	0.0	0.0	0.0
	*0.20*	25.3	24.5	22.8	25.1	0.0	0.0	0.0	0.0

**Table 4:** Average numbers of variables found in the simulation study for the noise-layer structure

δ_*i*_	Level	Number of variables found for the noise-layer structure			
		Noise Layer			
		BCS	FDR1	FDR2	STOC
*0*	*0.01*	19.5	0.0	0.0	9.6
	*0.03*	51.2	0.0	0.0	24.4
	*0.05*	70.7	0.1	0.0	33.5
	*0.07*	82.5	0.1	0.0	38.9
	*0.10*	92.8	0.2	0.0	45.0
	*0.15*	96.2	0.3	0.0	47.5
	*0.20*	99.3	0.4	0.0	50.0
*0.03*	*0.01*	15.4	0.0	0.0	4.5
	*0.03*	36.8	0.0	0.0	9.4
	*0.05*	49.0	0.0	0.0	12.6
	*0.07*	56.7	0.0	0.0	14.9
	*0.10*	64.7	0.0	0.0	17.5
	*0.15*	69.5	0.0	0.0	18.9
	*0.20*	71.0	0.0	0.0	20.0
*0.05*	*0.01*	12.1	0.0	0.0	0.0
	*0.03*	26.1	0.0	0.0	0.0
	*0.05*	36.0	0.0	0.0	0.0
	*0.07*	42.0	0.0	0.0	0.0
	*0.10*	46.8	0.0	0.0	0.0
	*0.15*	51.1	0.0	0.0	0.0
	*0.20*	52.8	0.0	0.0	0.0
*0.1*	*0.01*	0.0	0.0	0.0	0.0
	*0.03*	0.0	0.0	0.0	0.0
	*0.05*	0.0	0.0	0.0	0.0
	*0.07*	0.0	0.0	0.0	0.0
	*0.10*	0.0	0.0	0.0	0.0
	*0.15*	0.0	0.0	0.0	0.0
	*0.20*	0.0	0.0	0.0	0.0

**Table 5:** P-values and classification rates of logistic regression models by detected noise variables in the noise layers for the three-layer structure

δ_*i*_	Level	p-value				classification rate			
		BCS	FDR1	FDR2	STOC	BCS	FDR1	FDR2	STOC
*0*	*0.01*	0.0001	0.0001	–	–	0.6568	0.6600	–	–
	*0.03*	0.0002	0.0002	0.0002	0.0002	0.6578	0.6547	0.6526	0.6563
	*0.05*	0.0004	0.0004	0.0004	0.0004	0.6539	0.6508	0.6481	0.6532
	*0.07*	0.0005	0.0005	–	0.0005	0.6578	0.6537	–	0.6560
	*0.10*	0.0005	0.0005	0.0005	0.0005	0.6710	0.6669	0.6663	0.6691
	*0.15*	0.0004	0.0004	0.0004	0.0004	0.6769	0.6743	0.6739	0.6767
	*0.20*	0.0001	0.0001	0.0002	0.0001	0.6998	0.6979	0.6958	0.6996
*0.03*	*0.01*	0.0097	–	–	–	0.6200	–	–	–
	*0.03*	0.0006	–	–	0.0113	0.6450	–	–	0.6150
	*0.05*	0.0000	–	–	0.0015	0.7200	–	–	0.6300
	*0.07*	0.0000	–	–	0.0000	0.8200	–	–	0.7000
	*0.10*	0.0000	–	–	0.0000	0.8250	–	–	0.7100
	*0.15*	0.0000	–	–	0.0000	0.7900	–	–	0.6650
	*0.20*	0.0000	–	–	0.0000	0.8150	–	–	0.7700
*0.05*	*0.01*	0.0535	–	–	–	0.6000	–	–	–
	*0.03*	0.0003	–	–	–	0.6750	–	–	–
	*0.05*	0.0001	–	–	–	0.7150	–	–	–
	*0.07*	0.0000	–	–	–	0.6900	–	–	–
	*0.10*	0.0030	–	–	–	0.6600	–	–	–
	*0.15*	0.0000	–	–	–	0.7550	–	–	–
	*0.20*	0.0000	–	–	–	0.7950	–	–	–

The averaged numbers found by the various methods in the two-layer structure are presented in Table 5; For all levels, BCR still found more variables in layer 1 than STOCSYO and FDR. The BCR method also found more variables in layer 2 than STOCSYO and FDR. We also observe STOCSYO also detected more variables in layer 2 than FDR. The averaged numbers found by the various methods in the one-layer structure are presented in Table 1; BCR outperformed STOCSYO and FDR for all levels with regard to the number of variables found. The average number of variables in each layer filtered out by the BCS method in the layer structures along with the averaged p-values are presented in Additional files 4 and 5: Tables S6 and S7 according to the noise condition and tested level. Clearly, the number of the filtered variables increases as the noise conditions and levels increase. For example, the number of the filtered variables for levels ≤0.05 is quite much less than those for levels >0.05.It also increases as the structure moves from the three layers to the noise layers, which means increasing randomness. Consistently, most of the filtered variables appeared in the last noise layer, which practically demonstrates the use of the filtering step. For example, in the two-layer structure with noise condition 0 and level 0.10, the average number of the filtered variables in the noise layer is 64.69 while that in layer 1 is 0.52 and that in layer 2 is 10.18. Though small in number, the filtered variables in layer 1 partly explains the BCR never finds all 30 variables in layer 1.

**Fig. 4:**
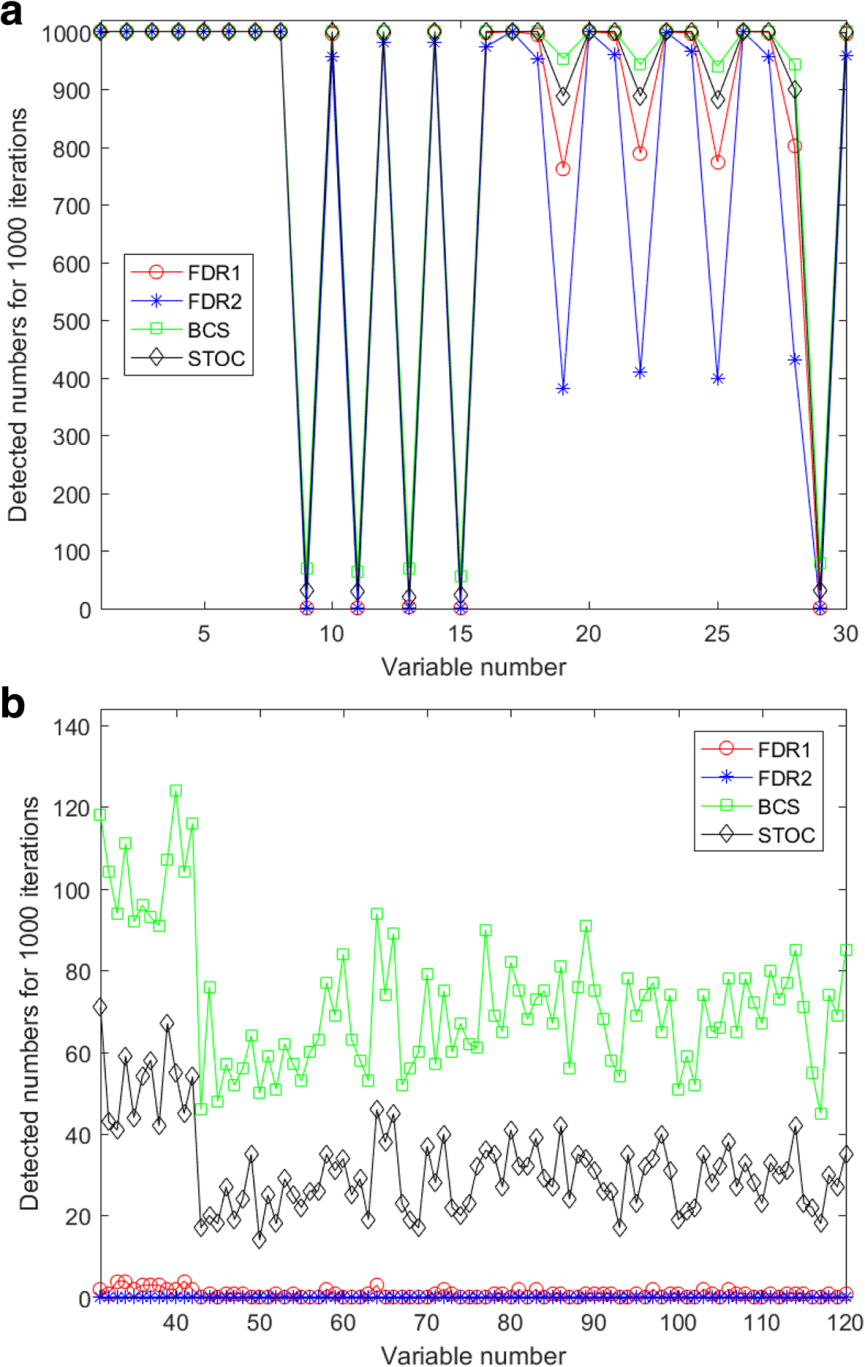
This figure illustrates selected variables by the four tested methods in the following conditions: **a** in layer 1 of the two-layer structure when noise condition *δ*_*i*_ = 0 and level = 0.05; **b** in layer 2 of the three-layer structure when noise condition *δ*_*i*_ = 0 and level = 0.10

Additionally, Fig. 4 and figures in Additional file 6: Figure S1 show the number of the selected variables by the four tested methods during the 1000 iterations in noise conditions *δ*_*i*_ = 0 or 0.05, levels 0.05 or 0.10, and three layer structures (one layer, two layers, and three layers). While the four methods repeatedly captured the eight strong variables, x_1_ to x_8_, in layer 1, as shown in Figs. 4 (a) and (b), the BCS method found variables, x_9_ to x_30_, in layer 1, as well as variables in layers 2 and 3, more frequently than the others. The tendency, however, weakens as the layer structure moves from the three-layer one to the one-layer one. Overall the FDR methods are strict in capturing variables, STOCSYO remains in between FDR and BCS, and BCS finds variables not only individually strong but also collectively separating.

### Application to real-life biological data

**Fig. 5:**
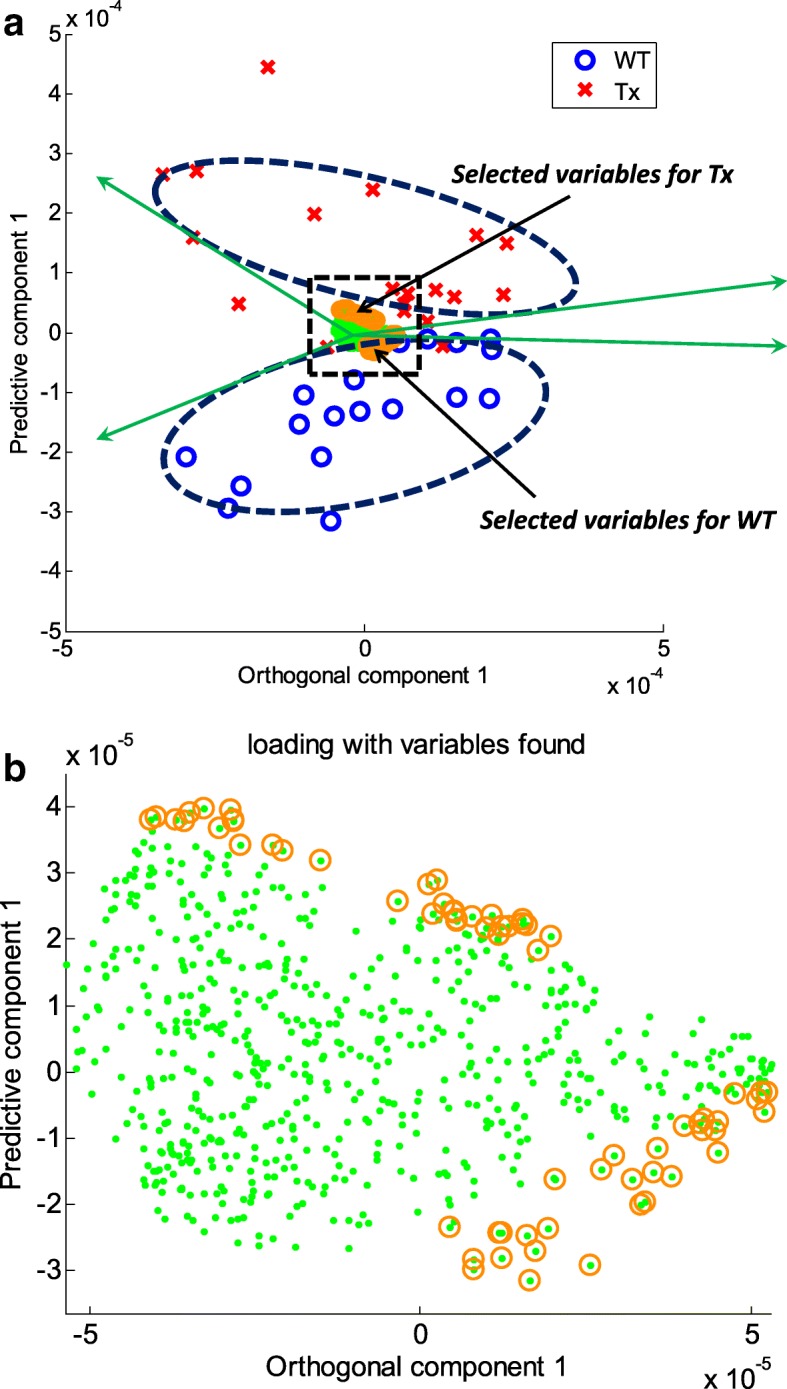
BCS analysis using orthogonal signal correction of high-resolution metabolomic data from liver mitochondria from thioredoxin-2 transgenic (TG) mice and wild-type (WT) littermate controls. **a** A two-dimensional score plot of orthogonal signal correction shows the first predictive component as a function of the first orthogonal component. **b** Corresponding loading plot, a zoomed-in version of the dotted-line rectangle of (**a**), with the top 5% of features to the separation of TG and WT metabolic profiles within the 95% correlation range contributing. A list of the 64 discriminatory m/z features identified is provided in Additional file 7: Table S1

To apply the proposed method, we examined a high-resolution metabolomics data set from a recent study of mitochondrial metabolomics of thioredoxin-2-overexpressing transgenic (TG) mice and wild-type (WT) littermate controls [30]. Thioredoxin (Trx2) is a small protein that regulates reduction-oxidation balance. The chosen dataset comprised anion exchange-high-resolution mass spectrometry data of mitochondria from 18 WT and 19 TG mice. Metabolic data were extracted from mass spectral analyses using apLCMS [10] and comprised high-resolution m/z features defined by m/z, retention time, and intensity. Each sample was analyzed in duplicate, and data for duplicates were averaged. Features with ≥30% missing values were excluded, resulting in 677 features for each sample. The included missing values were replaced by zero since no noticeable peaks at the m/z features were found as in [31]. Comparison of WT and TG data using FDR at q = 0.05 or q = 0.2 resulted in no features being detected as different. Similarly, STOCSYO detected no significant features. Application of BCR to identify features contributing to the separation of WT and TG mitochondria by the first orthogonal and the first predictive components from OPLS resulted in the identification of 64 features, as shown in Fig. 5 (See also Additional file 7: Table S1).

**Fig. 6:**
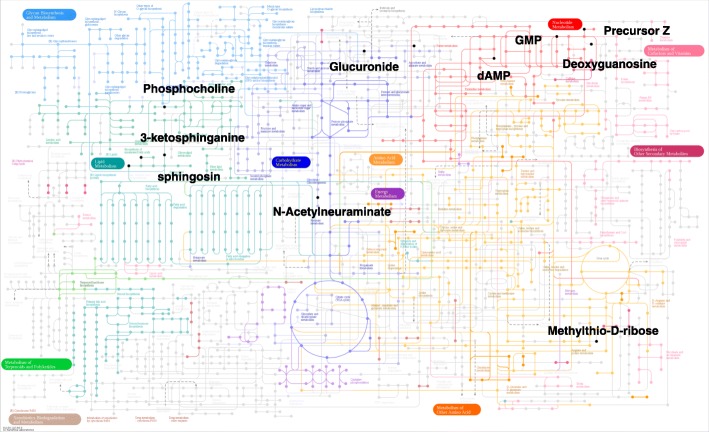
Kyoto Encyclopedia of Genes and Genomes (KEGG) pathway analysis of the 45 discriminatory features identified using the BCS approach. Forty-five discriminatory features are mapped onto 12 *Mus musculus* (mouse) KEGG metabolites (black dots). Those are involved in phosphotidylcholine, sphingosine, cysteine, methionine, and purine metabolism. The distributions suggest that multiple factors associated with Trx overexpression can be conceptualized using the group-wise feature selection provided by the BCS approach

As post-analysis we annotated the selected metabolites using the Metlin mass spectrometry database [32]. To determine the associated pathobiology, we applied KEGG (The Kyoto Encyclopedia of Genes and Genomes)-database pathway analysis [33]. Of the 64 features identified by BCR, the 45 had a variable importance projection score ≥ 1 [24]. These 45 discriminatory features were annotated in the Metlin database using only [M + H] + and [M + Na] + adducts (See Additional file 8: Table S2). Twelve of the 45 features were mapped using KEGG *Mus musculus* pathway analysis as shown in Fig.6.

More than two-thirds of the features did not match metabolites in the KEGG database, being considered as false positives in practice from the KEGG-database viewpoint. Phosphatidylcholine(18:3) at 518.32 m/z and phosphatidylcholine(18:2) at 520.34 m/z as well as choline phosphate at 184.07 m/z were increased in TG mitochondria compared to WT mitochondria as shown in Figs. 6 (a) and (d).

Phosphatidylcholine (PC) is one of the most abundant phospholipids as it forms part of the membrane bilayer. Hung et al. investigated the possible role of phosphatidylcholine supplementation as a way of slowing aging-related processes in senescence-accelerated mice [34]. In addition, Al-Orf found that excess and persistent intake of oxidized phosphatidylcholine caused significant damage to organs in male Wistar albino rats [35]. Thioredoxin overexpression in mice has been shown to attenuate oxidative stress [36].

**Fig. 7:**
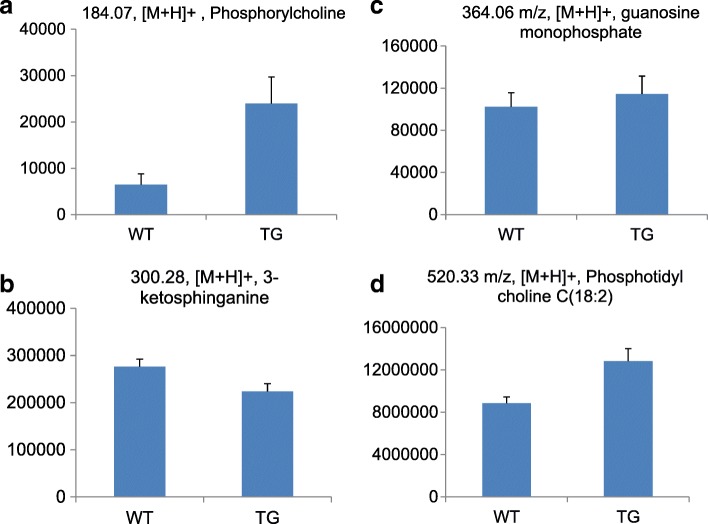
The relative concentrations of mitochondrial metabolites in WT and TG (**a**) phosphocholine, (**b**) 3-ketosphinganin, (**c**) guanosine monophosphate, (**d**) Phosphotidyl choline C(18:2)

The discovery of discriminating metabolites related to sphingosine was unanticipated but reasonable in terms of what is known about ceramide metabolism. Ceramide is an endogenous mediator of apoptotic cell death. For example, when the intracellular concentration of ceramide is elevated under oxidative stress, cellular proliferation is inhibited, and cellular apoptosis is induced [37]. Ceramide is synthesized at the endoplasmic reticulum from palmitoyl-CoA and serine, resulting in 3-ketosphinganine. The enzyme 3-ketosphinganine reductase generates sphinganine from 3-ketosphinganine. Sphinganine is acylated to dihydroceramide by sphinganine N-acyl-transferase. Finally, dihydroceramide is converted to ceramide by the activity of the dihydroceramide desaturase [38–40]. In this study, we observed a reduced amount of 3-ketosphinganine (300.28 m/z) in Trx2-overexpressing TG mice, suggesting that Trx2 decreases levels of 3-ketosphinganine, thereby conferring protection against apoptosis (Fig. 7 (b)). Thus, the discrimination of WT and TG mitochondria by 3-ketospingosine is consistent with available data on mitochondria, ceramide metabolism, and Trx2 protection against apoptosis signaling.

The discrimination of WT and TG mitochondria by guanosine monophosphate (GMP) at 364.06 m/z is also reasonable because GMP increases antioxidant function and attenuates oxidant cell death [41, 42]. Consistent with the anti-apoptotic effect of GMP, we observed increased GMP in Trx2 TG mice compared to that in WT mice, providing important evidence of overexpression of Trx2 (Fig. 7 (c)). The discrimination of WT and TG mitochondria by GMP is consistent with available data on mitochondria, the anti-apoptotic effect of GMP on oxidative stress, and Trx2 protection against apoptosis signaling.

Several methods currently exist to identify metabolites that are significantly different according to sample classification based upon the principles of FDR. Comparable methods to test for group behavior of metabolites in sample classification, however, do not exist. Data reduction methods are available to reduce a large number of variables into a smaller set of variables; this allows separation of classes according to the group behavior of metabolites. Previous graphical methods have resulted in identification of individual metabolites that contribute to group behavior; however, no criteria for inclusion or exclusion of metabolites were provided. Our newly developed method, BCR, uses statistical criteria for selection of metabolites contributing to group behavior. Evaluation of its performance with both simulated data and real data demonstrated its utility. The BCR method employs statistical principles to select variables that contribute group-wise to class discrimination. The method allows reproducible selection of metabolites that contribute to class separation, thereby facilitating practical developments in metabolomics research. Application of BCR could, in principle, provide a simple means to detect group-wise behavior of metabolites connected to different pathways and metabolic networks.

## Conclusions

We developed a dimensionality-reduction based approach termed a biplot correlation range that improves reliability of selection of metabolites contributing to group behavior for use in metabolic profiling applications for personalized medicine. Original variable interactions were used to assign scores according to group identity, and statistical principles were used to select variables in terms of increased score in the direction of a group identity within a correlation range. Testing by simulation and application to real data showed that this method improved selection of variables collectively responsible for group behavior. By providing a statistical basis differently from FDR and OPLS-coupled STOCSY approaches, the proposed method can reveal important metabolites that contribute to group behavior for analysis of complex metabolic data sets. As a future research direction, more rigorous add-on analysis of selected important metabolites such as the calculation of *p*-values by cross validation, sensitivity analysis of selection of components, and systemic post-analysis are in need of investigation.

## Additional files

Additional file 1: **Figure S1.** This figure illustrates selected variables by the four tested methods in the following conditions: (a) in layer 1 of the one-layer structure when noise condition δ_i=0.05 and level = 0.05; (b) in layer 2 of the two-layer structure when noise condition δ_i=0.05 and level = 0.05; (c) in layer 3 of the three-layer structure when noise condition δ_i=0 and level = 0.10; (d) in layer 3 of the two-layer structure when noise condition δ_i=0.05 and level = 0.10; (e) in the noise layer of the three-layer structure when noise condition δ_i=0 and level = 0.10; (f) in the noise layer of the one-layer structure when noise condition δ_i=0.05 and level = 0.05. (DOCX 16 kb)

Additional file 2: **Table S1.** High-resolution metabolomics features discriminating liver mitochondria from thioredoxin-2 transgenic mice from wildtype littermates as identified by PCLS. (DOCX 16 kb)

Additional file 3: **Table S2.** Three hundred ten features with variable importance projection (VIP) score greater than and equal to 1 were listed from mitochondria between wild and thioredoxin-2 transgenic mice. (DOCX 16 kb)

Additional file 4: **Table S3.** *P*-values and classification rates of logistic regression models by detected noise variables in the noise layers for the two-layer structure. (DOCX 19 kb)

Additional file 5: **Table S4.** *P*-values and classification rates of logistic regression models by detected noise variables in the noise layers for the one-layer structure. (DOCX 16 kb)

Additional file 6: **Table S5.** *P*-values and classification rates of logistic regression models by detected noise variables in the noise layers for the noise-layer structure. (DOCX 2123 kb)

Additional file 7: **Table S6.** The average number of filtered variables in each layer and the averaged P-values for the three-layer and two-layer structures from the BCS method. (DOC 75 kb)

Additional file 8: **Table S7.** The average number of filtered variables in each layer and the averaged P-values for the one-layer and noise-layer structures from the BCS method. (DOCX 58 kb)
