# Supplementary material for: A biplot correlation range for group-wise metabolite selection in mass spectrometry
Source: BioData Min. 2019 Feb 4;12:4. doi: 10.1186/s13040-019-0191-2 (PMC6360680; doi:10.1186/s13040-019-0191-2)
Supplement: Supplementary file 1 — Figure S1. This figure illustrates selected variables by the four tested methods in the following conditions: (a) in layer 1 of the one-layer structure when noise condition δ_i=0.05 and level = 0.05; (b) in layer 2 of the two-layer structure when noise condition δ_i=0.05 and level = 0.05; (c) in layer 3 of the three-layer structure when noise condition δ_i=0 and level = 0.10; (d) in layer 3 of the two-layer structure when noise condition δ_i=0.05 and level = 0.10; (e) in the noise layer of the three-layer structure when noise condition δ_i=0 and level = 0.10; (f) in the noise layer of the one-layer structure when noise condition δ_i=0.05 and level = 0.05. (DOCX 16 kb) [file 13040_2019_191_MOESM1_ESM.docx]

Additional file 1: Table S3. P-values and classification rates of logistic regression models by detected noise variables in the noise layers for the two-layer structure

| $\delta_{i}$ | Level | p-value | | | | classification rate | | | |
| --- | --- | --- | --- | --- | --- | --- | --- | --- | --- |
|  |  | BCS | FDR1 | FDR2 | STOC | BCS | FDR1 | FDR2 | STOC |
| *0* | *0.01* | 0.0001 | 0.0001 | 0.0001 | - | 0.6570 | 0.6563 | 0.6675 | - |
|  | *0.03* | 0.0002 | 0.0002 | 0.0002 | 0.0002 | 0.6606 | 0.6557 | 0.6561 | 0.6568 |
|  | *0.05* | 0.0003 | 0.0003 | 0.0003 | 0.0003 | 0.6679 | 0.6625 | 0.6631 | 0.6656 |
|  | *0.07* | 0.0003 | 0.0003 | 0.0003 | 0.0003 | 0.6701 | 0.6660 | 0.6670 | 0.6685 |
|  | *0.10* | 0.0004 | 0.0004 | 0.0004 | 0.0004 | 0.6821 | 0.6784 | 0.6766 | 0.6821 |
|  | *0.15* | 0.0002 | 0.0002 | 0.0002 | 0.0002 | 0.6992 | 0.6960 | 0.6969 | 0.6986 |
|  | *0.20* | 0.0002 | 0.0002 | 0.0002 | 0.0002 | 0.7285 | 0.7258 | 0.7271 | 0.7276 |
| *0.03* | *0.01* | 0.0412 | - | - | - | 0.5750 | - | - | - |
|  | *0.03* | 0.0000 | - | - | 0.0020 | 0.7750 | - | - | 0.6400 |
|  | *0.05* | 0.0000 | - | - | 0.0000 | 0.8300 | - | - | 0.6900 |
|  | *0.07* | 0.0000 | - | - | 0.0000 | 0.8800 | - | - | 0.7400 |
|  | *0.10* | 0.0000 | - | - | 0.0001 | 0.8200 | - | - | 0.7050 |
|  | *0.15* | 0.0000 | - | - | 0.0000 | 1.0000 | - | - | 0.7600 |
|  | *0.20* | 0.0000 | - | - | 0.0000 | 0.9450 | - | - | 0.8300 |
| *0.05* | *0.01* | 0.0352 | - | - | - | 0.6150 | - | - | - |
|  | *0.03* | 0.0003 | - | - | - | 0.7300 | - | - | - |
|  | *0.05* | 0.0000 | - | - | - | 0.8300 | - | - | - |
|  | *0.07* | 0.0000 | - | - | - | 0.8150 | - | - | - |
|  | *0.10* | 0.0000 | - | - | - | 0.8150 | - | - | - |
|  | *0.15* | 0.0000 | - | - | - | 0.8950 | - | - | - |
|  | *0.20* | 0.0000 | - | - | - | 0.8300 | - | - | - |
