# Supplementary material for: A biplot correlation range for group-wise metabolite selection in mass spectrometry
Source: BioData Min. 2019 Feb 4;12:4. doi: 10.1186/s13040-019-0191-2 (PMC6360680; doi:10.1186/s13040-019-0191-2)
Supplement: Supplementary file 2 — Table S1. High-resolution metabolomics features discriminating liver mitochondria from thioredoxin-2 transgenic mice from wildtype littermates as identified by PCLS. (DOCX 16 kb) [file 13040_2019_191_MOESM2_ESM.docx]

Additional file 2: Table S4. P-values and classification rates of logistic regression models by detected noise variables in the noise layers for the one-layer structure

| $\delta_{i}$ | Level | p-value | | | | classification rate | | | |
| --- | --- | --- | --- | --- | --- | --- | --- | --- | --- |
|  |  | BCS | FDR1 | FDR2 | STOC | BCS | FDR1 | FDR2 | STOC |
| *0* | *0.01* | 0.0001 | 0.0001 | 0.0001 | - | 0.6361 | 0.6368 | 0.6389 | - |
|  | *0.03* | 0.0002 | 0.0002 | 0.0001 | 0.0002 | 0.6382 | 0.6336 | 0.6354 | 0.6348 |
|  | *0.05* | 0.0003 | 0.0003 | 0.0003 | 0.0003 | 0.6463 | 0.6412 | 0.6381 | 0.6444 |
|  | *0.07* | 0.0002 | 0.0002 | 0.0002 | 0.0002 | 0.6579 | 0.6540 | 0.6530 | 0.6571 |
|  | *0.10* | 0.0003 | 0.0003 | 0.0003 | 0.0003 | 0.6634 | 0.6597 | 0.6602 | 0.6624 |
|  | *0.15* | 0.0003 | 0.0003 | 0.0002 | 0.0003 | 0.6875 | 0.6842 | 0.6840 | 0.6864 |
|  | *0.20* | 0.0001 | 0.0001 | 0.0001 | 0.0001 | 0.7238 | 0.7210 | 0.7205 | 0.7229 |
| *0.03* | *0.01* | 0.0199 | - | - | - | 0.6100 | - | - | - |
|  | *0.03* | 0.0000 | - | - | 0.0077 | 0.8350 | - | - | 0.6500 |
|  | *0.05* | 0.0000 | - | - | 0.0000 | 0.8850 | - | - | 0.7450 |
|  | *0.07* | 0.0000 | - | - | 0.0000 | 1.0000 | - | - | 0.7600 |
|  | *0.10* | 0.0000 | - | - | 0.0000 | 1.0000 | - | - | 0.7500 |
|  | *0.15* | 0.0000 | - | - | 0.0000 | 0.9550 | - | - | 0.7350 |
|  | *0.20* | 0.0000 | - | - | 0.0000 | 0.9150 | - | - | 0.7500 |
| *0.05* | *0.01* | 0.0194 | - | - | - | 0.5900 | - | - | - |
|  | *0.03* | 0.0000 | - | - | - | 0.7850 | - | - | - |
|  | *0.05* | 0.0000 | - | - | - | 0.8200 | - | - | - |
|  | *0.07* | 0.0000 | - | - | - | 0.8650 | - | - | - |
|  | *0.10* | 0.0000 | - | - | - | 0.8700 | - | - | - |
|  | *0.15* | 0.0000 | - | - | - | 0.8400 | - | - | - |
|  | *0.20* | 0.0000 | - | - | - | 0.9150 | - | - | - |
