# Supplementary material for: A biplot correlation range for group-wise metabolite selection in mass spectrometry
Source: BioData Min. 2019 Feb 4;12:4. doi: 10.1186/s13040-019-0191-2 (PMC6360680; doi:10.1186/s13040-019-0191-2)
Supplement: Supplementary file 3 — Table S2. Three hundred ten features with variable importance projection (VIP) score greater than and equal to 1 were listed from mitochondria between wild and thioredoxin-2 transgenic mice. (DOCX 16 kb) [file 13040_2019_191_MOESM3_ESM.docx]

Additional file 3: Table S5. P-values and classification rates of logistic regression models by detected noise variables in the noise layers for the noise-layer structure

| $\delta_{i}$ | Level | p-value | | | | classification rate | | | |
| --- | --- | --- | --- | --- | --- | --- | --- | --- | --- |
|  |  | BCS | FDR1 | FDR2 | STOC | BCS | FDR1 | FDR2 | STOC |
| *0* | *0.01* | 0.0000 | - | - | 0.0000 | 0.7950 | - | - | 0.7550 |
|  | *0.03* | 0.0000 | 0.0000 | - | 0.0000 | 0.7025 | 0.6283 | - | 0.6738 |
|  | *0.05* | 0.0000 | 0.0000 | - | 0.0000 | 0.7079 | 0.6592 | - | 0.6914 |
|  | *0.07* | 0.0000 | 0.0000 | - | 0.0000 | 0.6919 | 0.6479 | - | 0.6819 |
|  | *0.10* | 0.0000 | 0.0000 | - | 0.0000 | 0.6838 | 0.6575 | - | 0.6758 |
|  | *0.15* | 0.0000 | 0.0000 | - | 0.0000 | 0.6792 | 0.6603 | - | 0.6792 |
|  | *0.20* | 0.0000 | 0.0000 | - | 0.0000 | 0.6776 | 0.6630 | - | 0.6770 |
| *0.03* | *0.01* | 0.0004 | - | - | 0.0033 | 0.6850 | - | - | 0.6300 |
|  | *0.03* | 0.0000 | - | - | 0.0000 | 0.8150 | - | - | 0.6800 |
|  | *0.05* | 0.0000 | - | - | 0.0000 | 0.8400 | - | - | 0.7150 |
|  | *0.07* | 0.0000 | - | - | 0.0000 | 0.9150 | - | - | 0.6900 |
|  | *0.10* | 0.0000 | - | - | 0.0000 | 0.9200 | - | - | 0.7350 |
|  | *0.15* | 0.0000 | - | - | 0.0000 | 1.0000 | - | - | 0.7700 |
|  | *0.20* | 0.0000 | - | - | 0.0000 | 1.0000 | - | - | 0.7200 |
| *0.05* | *0.01* | 0.0009 | - | - | - | 0.6750 | - | - | - |
|  | *0.03* | 0.0001 | - | - | - | 0.7550 | - | - | - |
|  | *0.05* | 0.0000 | - | - | - | 0.7850 | - | - | - |
|  | *0.07* | 0.0000 | - | - | - | 0.8500 | - | - | - |
|  | *0.10* | 0.0000 | - | - | - | 0.8550 | - | - | - |
|  | *0.15* | 0.0000 | - | - | - | 0.8900 | - | - | - |
|  | *0.20* | 0.0000 | - | - | - | 0.9100 | - | - | - |
