# Supplementary material for: A biplot correlation range for group-wise metabolite selection in mass spectrometry
Source: BioData Min. 2019 Feb 4;12:4. doi: 10.1186/s13040-019-0191-2 (PMC6360680; doi:10.1186/s13040-019-0191-2)
Supplement: Supplementary file 4 — Table S3. P-values and classification rates of logistic regression models by detected noise variables in the noise layers for the two-layer structure. (DOCX 19 kb) [file 13040_2019_191_MOESM4_ESM.docx]

Additional file 4: Table S6. The average number of filtered variables in each layer and the averaged P-values for the three-layer and two-layer structures from the BCS method

| $\delta_{i}$ | Level | Three Layers | | | | | Two Layers | | | |
| --- | --- | --- | --- | --- | --- | --- | --- | --- | --- | --- |
|  |  | p-value avg. | num. in layer 1 | num. in layer 2 | num. in layer 3 | num. in noise layer | p-value avg. | num. in layer 1 | num. in layer 2 | num. in noise layer |
| *0* | *0.01* | - | 0.00 | 0.00 | 0.00 | 0.00 | - | 0.00 | 0.00 | 0.00 |
|  | *0.03* | 0.137 | 0.01 | 0.46 | 1.12 | 0.24 | 0.117 | 0.01 | 0.05 | 0.25 |
|  | *0.05* | 0.152 | 0.10 | 2.85 | 6.59 | 2.80 | 0.126 | 0.10 | 0.93 | 5.11 |
|  | *0.07* | 0.163 | 0.20 | 6.26 | 15.92 | 9.48 | 0.139 | 0.24 | 3.83 | 23.20 |
|  | *0.10* | 0.181 | 0.39 | 12.34 | 33.50 | 24.58 | 0.162 | 0.52 | 10.18 | 64.69 |
|  | *0.15* | 0.215 | 0.56 | 22.49 | 66.33 | 53.67 | 0.205 | 0.94 | 21.63 | 142.7 |
|  | *0.20* | 0.248 | 0.77 | 30.74 | 96.88 | 80.78 | 0.25 | 1.26 | 31.31 | 217.0 |
| *0.03* | *0.01* | 0.126 | 0.00 | 0.01 | 0.06 | 0.00 | 0.112 | 0.00 | 0.00 | 0.01 |
|  | *0.03* | 0.141 | 0.05 | 1.13 | 2.55 | 0.68 | 0.122 | 0.05 | 0.40 | 2.06 |
|  | *0.05* | 0.157 | 0.16 | 4.01 | 10.35 | 5.27 | 0.133 | 0.18 | 2.49 | 14.80 |
|  | *0.07* | 0.17 | 0.22 | 7.96 | 20.73 | 13.48 | 0.147 | 0.39 | 6.21 | 39.06 |
|  | *0.10* | 0.187 | 0.39 | 14.32 | 39.37 | 29.98 | 0.171 | 0.66 | 13.00 | 84.45 |
|  | *0.15* | 0.221 | 0.79 | 23.71 | 70.92 | 62.20 | 0.214 | 1.05 | 23.77 | 161.9 |
|  | *0.20* | 0.253 | 0.94 | 32.10 | 100.0 | 88.80 | 0.257 | 1.42 | 32.78 | 237.6 |
| *0.05* | *0.01* | 0.129 | 0.00 | 0.02 | 0.06 | 0.01 | 0.114 | 0.00 | 0.01 | 0.04 |
|  | *0.03* | 0.146 | 0.05 | 1.63 | 4.16 | 1.44 | 0.126 | 0.10 | 1.01 | 5.46 |
|  | *0.05* | 0.163 | 0.16 | 5.17 | 13.43 | 7.35 | 0.138 | 0.26 | 3.92 | 23.97 |
|  | *0.07* | 0.173 | 0.28 | 9.22 | 24.52 | 17.44 | 0.153 | 0.44 | 8.10 | 51.74 |
|  | *0.10* | 0.192 | 0.46 | 15.44 | 42.88 | 34.03 | 0.177 | 0.69 | 14.90 | 98.56 |
|  | *0.15* | 0.224 | 0.66 | 24.98 | 75.25 | 64.37 | 0.22 | 1.11 | 25.15 | 175.9 |
|  | *0.20* | 0.255 | 0.87 | 32.38 | 103.9 | 92.17 | 0.262 | 1.45 | 33.75 | 249.2 |
| *0.1* | *0.01* | 0.13 | 0.00 | 0.04 | 0.07 | 0.02 | 0.121 | 0.00 | 0.10 | 0.63 |
|  | *0.03* | 0.154 | 0.09 | 2.76 | 6.72 | 3.13 | 0.138 | 0.28 | 3.54 | 22.63 |
|  | *0.05* | 0.168 | 0.26 | 7.57 | 19.70 | 13.25 | 0.153 | 0.45 | 8.14 | 55.48 |
|  | *0.07* | 0.181 | 0.38 | 11.92 | 31.98 | 25.44 | 0.169 | 0.63 | 12.75 | 87.70 |
|  | *0.10* | 0.2 | 0.59 | 18.82 | 52.22 | 43.94 | 0.193 | 0.92 | 19.01 | 136.0 |
|  | *0.15* | 0.232 | 0.68 | 27.10 | 82.80 | 74.77 | 0.234 | 1.17 | 28.49 | 212.8 |
|  | *0.20* | 0.264 | 0.99 | 34.47 | 110.4 | 108.3 | 0.274 | 1.52 | 36.43 | 284.7 |
