# Supplementary material for: A biplot correlation range for group-wise metabolite selection in mass spectrometry
Source: BioData Min. 2019 Feb 4;12:4. doi: 10.1186/s13040-019-0191-2 (PMC6360680; doi:10.1186/s13040-019-0191-2)
Supplement: Supplementary file 5 — Table S4. P-values and classification rates of logistic regression models by detected noise variables in the noise layers for the one-layer structure. (DOCX 16 kb) [file 13040_2019_191_MOESM5_ESM.docx]

Additional file 5: Table S7. The average number of filtered variables in each layer and the averaged P-values for the one-layer and noise-layer structures from the BCS method

| $\delta_{i}$ | Level | One layer | | | Noise Layer | |
| --- | --- | --- | --- | --- | --- | --- |
|  |  | p-value avg. | num. in layer 1 | num. in noise layer | p-value avg. | num. in noise layer |
| *0* | *0.01* | - | 0.00 | 0.00 | 0.131 | 0.47 |
|  | *0.03* | 0.114 | 0.00 | 0.18 | 0.155 | 9.10 |
|  | *0.05* | 0.123 | 0.08 | 5.36 | 0.175 | 29.44 |
|  | *0.07* | 0.137 | 0.22 | 25.49 | 0.194 | 57.64 |
|  | *0.10* | 0.159 | 0.47 | 74.82 | 0.222 | 108.30 |
|  | *0.15* | 0.207 | 0.96 | 169.1 | 0.269 | 200.8 |
|  | *0.20* | 0.255 | 1.33 | 254.5 | 0.316 | 297.9 |
| *0.03* | *0.01* | - | 0.00 | 0.00 | 0.155 | 4.10 |
|  | *0.03* | 0.119 | 0.04 | 2.37 | 0.174 | 22.81 |
|  | *0.05* | 0.131 | 0.25 | 17.50 | 0.194 | 49.56 |
|  | *0.07* | 0.146 | 0.29 | 46.45 | 0.211 | 81.65 |
|  | *0.10* | 0.171 | 0.64 | 99.86 | 0.24 | 134.2 |
|  | *0.15* | 0.217 | 1.13 | 191.3 | 0.287 | 230.8 |
|  | *0.20* | 0.264 | 1.54 | 279.7 | 0.332 | 327.8 |
| *0.05* | *0.01* | 0.117 | 0.00 | 0.10 | 0.16 | 8.11 |
|  | *0.03* | 0.125 | 0.14 | 6.91 | 0.185 | 33.46 |
|  | *0.05* | 0.137 | 0.28 | 29.80 | 0.203 | 63.90 |
|  | *0.07* | 0.153 | 0.54 | 62.68 | 0.224 | 98.46 |
|  | *0.10* | 0.178 | 0.68 | 118.9 | 0.252 | 151.8 |
|  | *0.15* | 0.225 | 1.16 | 209.3 | 0.296 | 249.0 |
|  | *0.20* | 0.268 | 1.46 | 295.8 | 0.34 | 347.6 |
| *0.1* | *0.01* | 0.12 | 0.00 | 2.10 | 0.18 | 20.00 |
|  | *0.03* | 0.139 | 0.29 | 33.68 | 0.206 | 60.00 |
|  | *0.05* | 0.154 | 0.48 | 72.31 | 0.229 | 100.0 |
|  | *0.07* | 0.17 | 0.69 | 109.8 | 0.249 | 139.9 |
|  | *0.10* | 0.196 | 0.91 | 165.8 | 0.277 | 199.9 |
|  | *0.15* | 0.24 | 1.38 | 255.5 | 0.319 | 299.8 |
|  | *0.20* | 0.282 | 1.76 | 340.2 | 0.36 | 399.6 |
