# Supplementary material for: A biplot correlation range for group-wise metabolite selection in mass spectrometry
Source: BioData Min. 2019 Feb 4;12:4. doi: 10.1186/s13040-019-0191-2 (PMC6360680; doi:10.1186/s13040-019-0191-2)

**Additional file 6: Figure S1.** This figure illustrates selected variables by the four tested methods in the following conditions: (a) in layer 1 of the one-layer structure when noise condition $\delta_{i}=0.05$ and level = $0.05$; (b) in layer 2 of the two-layer structure when noise condition $\delta_{i}=0.05$ and level = $0.05$; (c) in layer 3 of the three-layer structure when noise condition $\delta_{i}=0$ and level = $0.10$; (d) in layer 3 of the two-layer structure when noise condition $\delta_{i}=0.05$ and level = $0.10$; (e) in the noise layer of the three-layer structure when noise condition $\delta_{i}=0$ and level = $0.10$; (f) in the noise layer of the one-layer structure when noise condition $\delta_{i}=0.05$ and level = $0.05$;

(a)


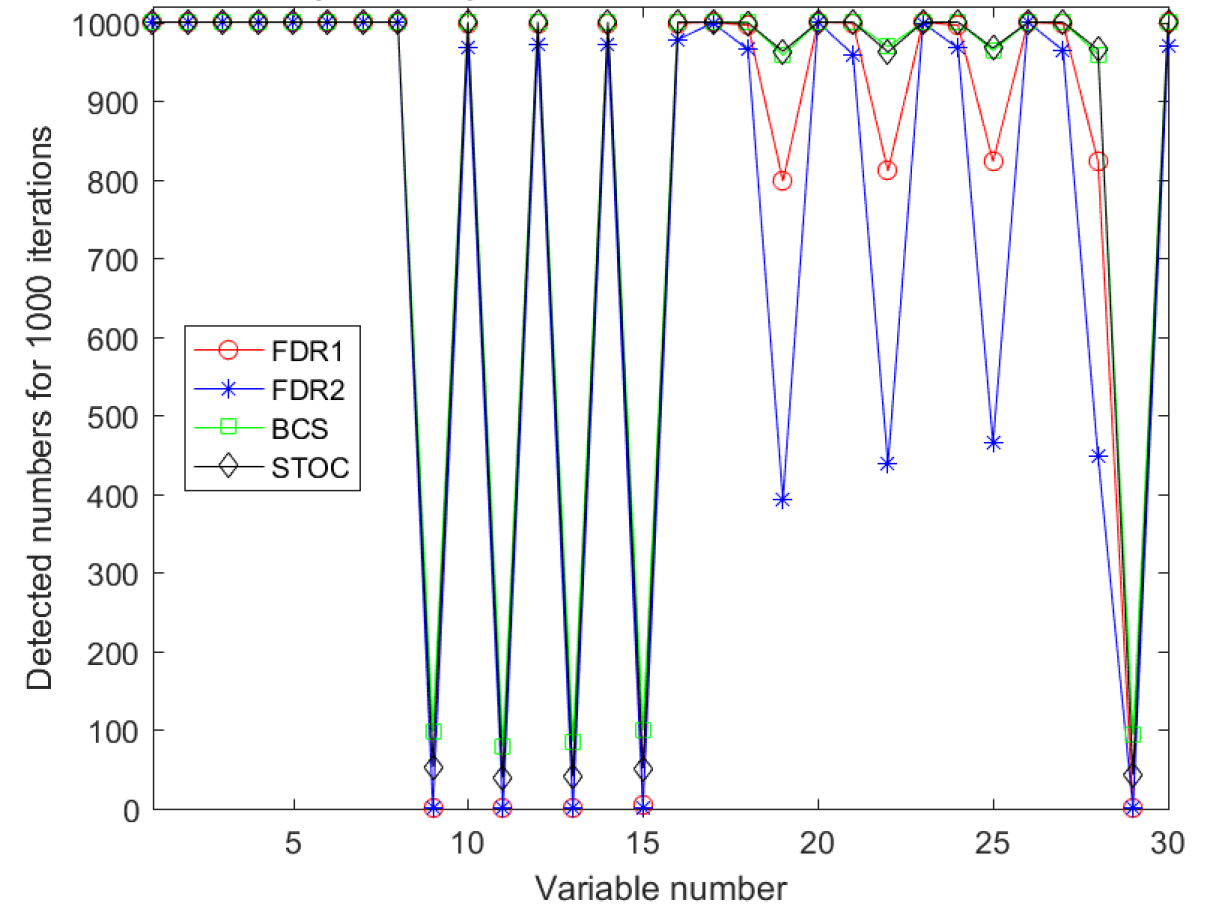


(b)


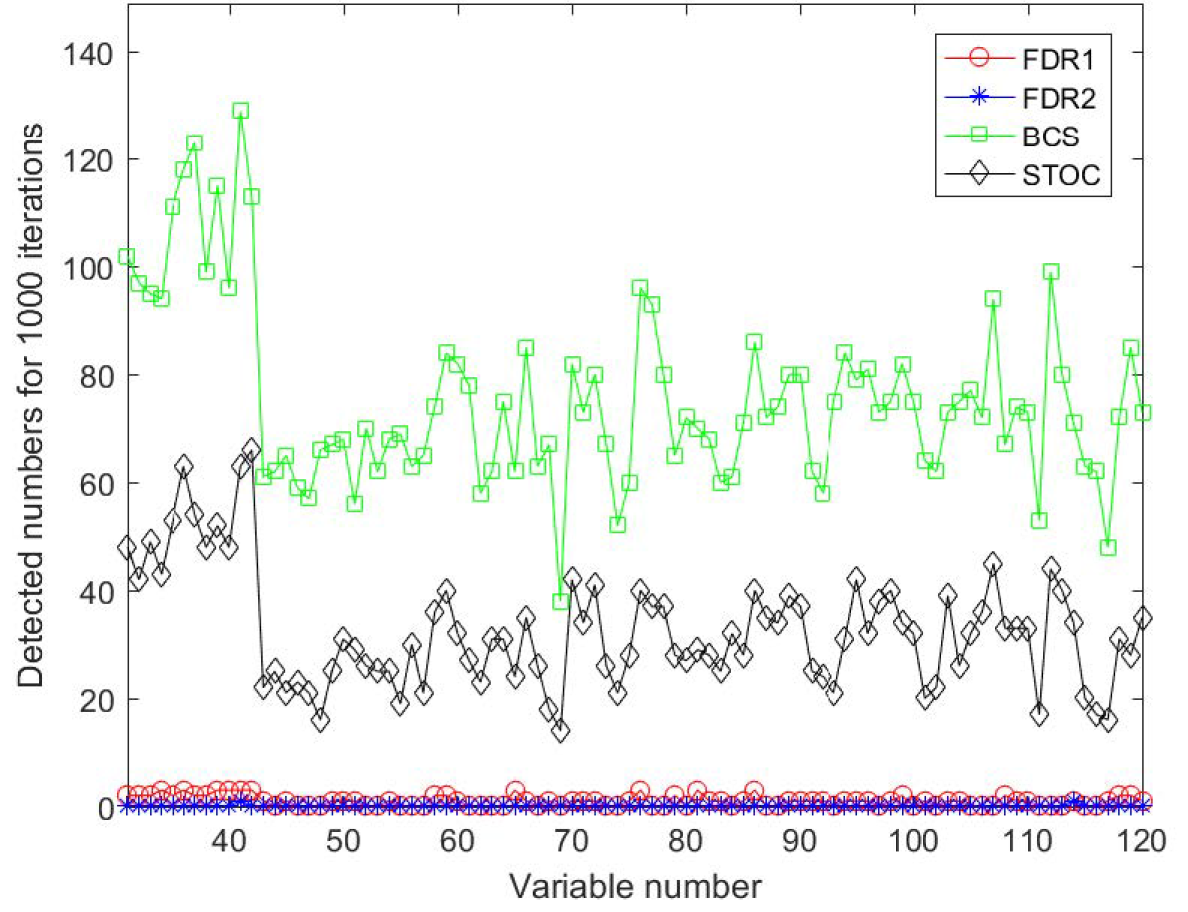


(c)


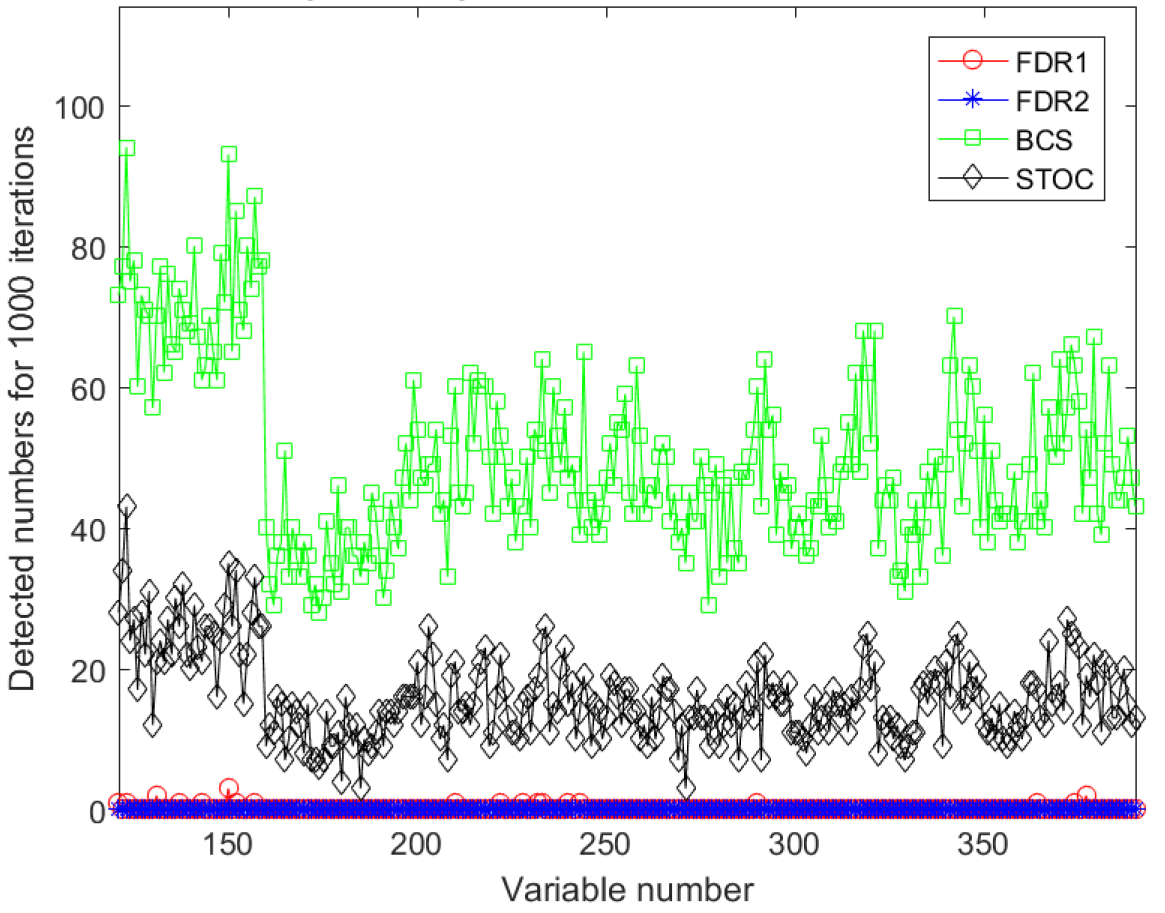


(d)


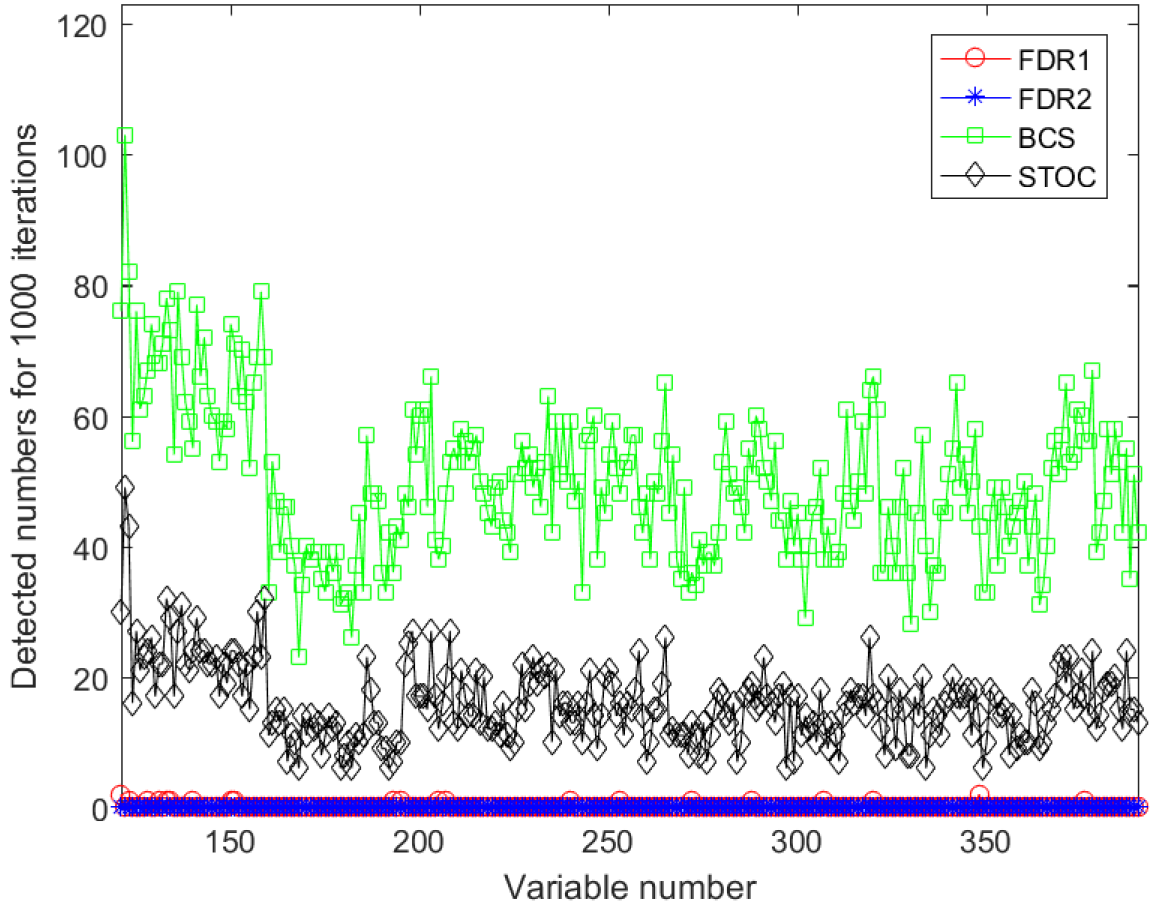


(e)


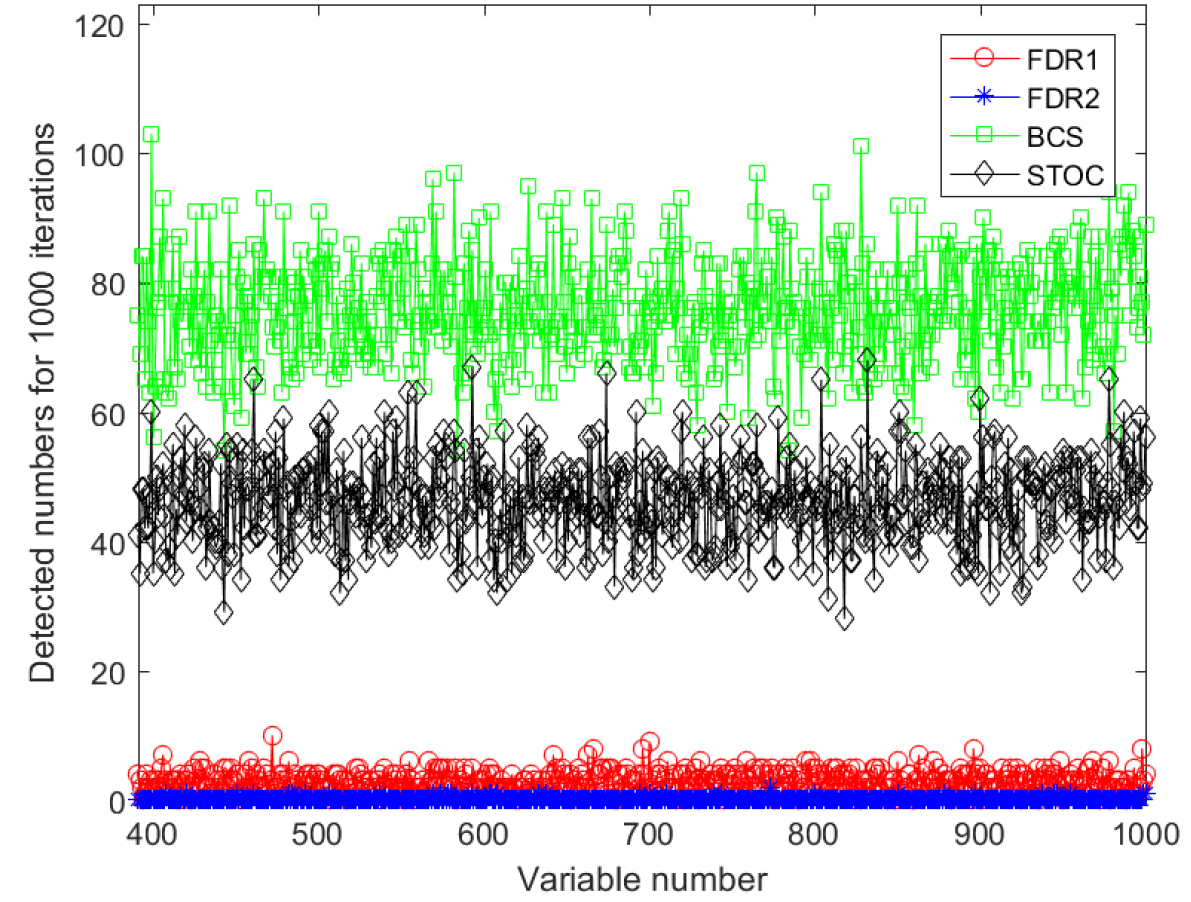


(f)


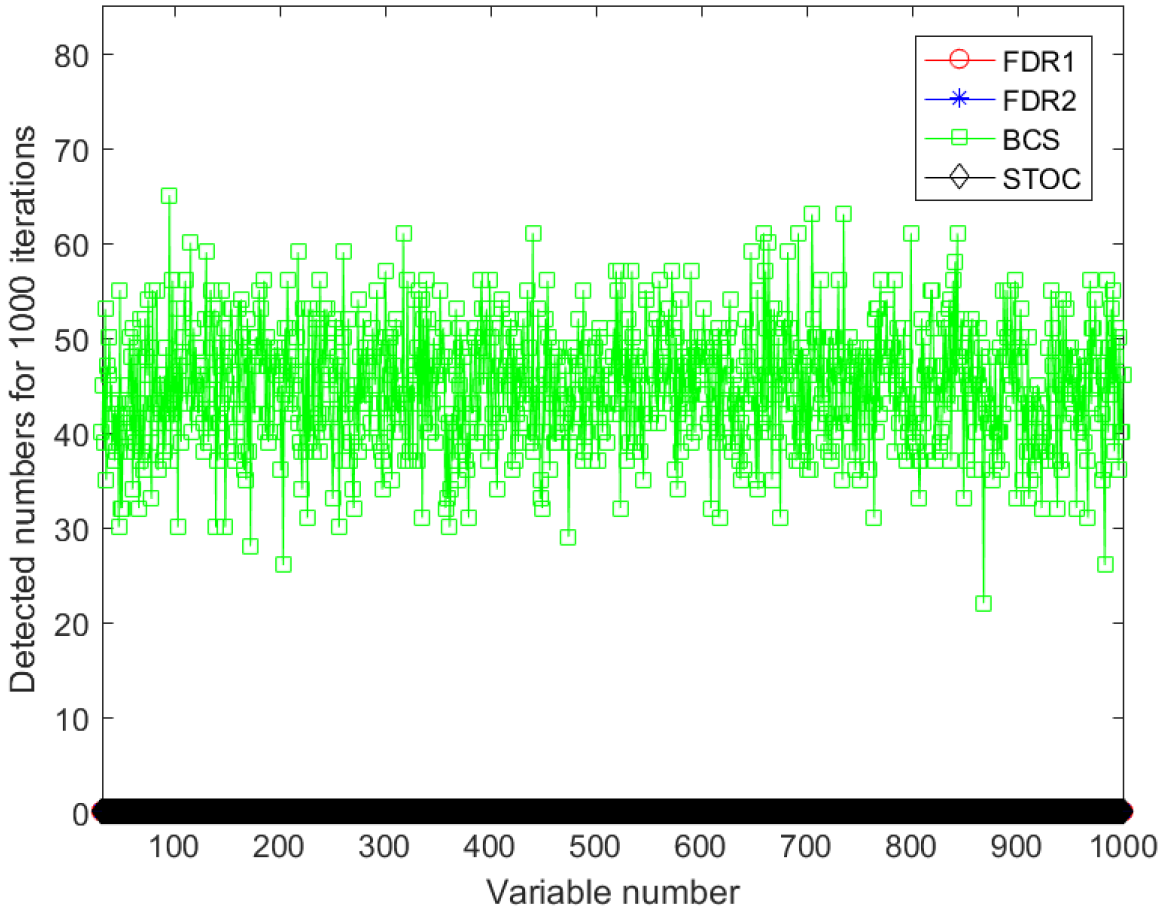

Supplement: Supplementary file 6 — Table S5. P-values and classification rates of logistic regression models by detected noise variables in the noise layers for the noise-layer structure. (DOCX 2123 kb) [file 13040_2019_191_MOESM6_ESM.docx]
