# Supplementary material for: A biplot correlation range for group-wise metabolite selection in mass spectrometry
Source: BioData Min. 2019 Feb 4;12:4. doi: 10.1186/s13040-019-0191-2 (PMC6360680; doi:10.1186/s13040-019-0191-2)
Supplement: Supplementary file 7 — Table S6. The average number of filtered variables in each layer and the averaged P-values for the three-layer and two-layer structures from the BCS method. (DOC 75 kb) [file 13040_2019_191_MOESM7_ESM.doc]

**Additional file 7: Table S1. High-resolution metabolomics features discriminating liver mitochondria from thioredoxin-2 transgenic mice from wildtype littermates as identified by PCLS**

|  |  | **Intensity** | |  |  |  | **Intensity** | |  |
| --- | --- | --- | --- | --- | --- | --- | --- | --- | --- |
| **m/z** | **RT** | **Wt_avg** | **Tg_avg** | **p-value** | **m/z** | **RT** | **Wt_avg** | **Tg_avg** | **p-value** |
| 168.069 | 76 | 154349 | 151672 | 0.895852 | 389.173 | 353 | 19696 | 15094 | 0.458516 |
| 184.064 | 79 | 41626 | 38961 | 0.721298 | 398.999 | 536 | 12518 | 28947 | 0.088315 |
| 184.073 | 400 | 4332 | 20191 | 0.013427 | 408.088 | 548 | 47891 | 39033 | 0.425332 |
| 196.100 | 76 | 153788 | 145151 | 0.724792 | 408.291 | 373 | 13358 | 26773 | 0.061692 |
| 198.079 | 78 | 43700 | 40422 | 0.722558 | 422.001 | 538 | 100658 | 158879 | 0.101882 |
| 206.071 | 76 | 64842 | 76138 | 0.007312 | 426.166 | 458 | 292793 | 333906 | 0.080599 |
| 212.058 | 168 | 1619822 | 1558589 | 0.736668 | 427.106 | 160 | 11162 | 31910 | 0.027573 |
| 213.061 | 162 | 74090 | 66024 | 0.471957 | 467.531 | 555 | 45826 | 36243 | 0.38136 |
| 228.072 | 77 | 63537 | 59356 | 0.716021 | 469.121 | 162 | 19149 | 37017 | 0.070189 |
| 244.100 | 76 | 281454 | 289205 | 0.883362 | 470.158 | 77 | 672325 | 757845 | 0.098324 |
| 244.138 | 165 | 11276 | 12311 | 0.802013 | 471.164 | 76 | 64069 | 73365 | 0.109687 |
| 268.103 | 75 | 247967 | 248668 | 0.990618 | 477.176 | 76 | 387435 | 389078 | 0.985946 |
| 272.945 | 60 | 214252 | 227378 | 0.470028 | 485.292 | 58 | 65935 | 110666 | 0.172299 |
| 278.105 | 326 | 28529 | 16034 | 0.031167 | 486.134 | 76 | 83288 | 94602 | 0.0846 |
| 279.646 | 416 | 16940 | 48368 | 0.004348 | 499.072 | 198 | 72781 | 64772 | 0.362884 |
| 290.133 | 158 | 53390 | 94083 | 0.023675 | 514.335 | 69 | 137859 | 152148 | 0.348711 |
| 300.288 | 146 | 276231 | 223921 | 0.029109 | 518.321 | 308 | 54780 | 96602 | 0.024069 |
| 310.112 | 553 | 221127 | 193256 | 0.368029 | 520.336 | 479 | 8856782 | 12816440 | 0.006023 |
| 311.100 | 463 | 725475 | 701763 | 0.856215 | 521.339 | 479 | 2298035 | 3335008 | 0.00764 |
| 312.103 | 446 | 47552 | 35945 | 0.318862 | 527.637 | 170 | 13509 | 13074 | 0.90686 |
| 319.953 | 512 | 233196 | 362232 | 0.219015 | 542.322 | 316 | 924188 | 1304171 | 0.031163 |
| 319.953 | 27 | 401657 | 486842 | 0.422662 | 544.739 | 558 | 22706 | 8972 | 0.065303 |
| 326.106 | 555 | 953024 | 769965 | 0.149116 | 544.897 | 61 | 381841 | 392567 | 0.784458 |
| 327.110 | 554 | 92662 | 68473 | 0.073237 | 568.335 | 505 | 3370588 | 4202839 | 0.057037 |
| 332.074 | 545 | 25290 | 16586 | 0.196461 | 569.338 | 501 | 972097 | 1224838 | 0.057192 |
| 332.082 | 512 | 294261 | 369245 | 0.398267 | 590.317 | 458 | 36064 | 128164 | 0.02697 |
| 348.273 | 424 | 74578 | 40652 | 0.090642 | 599.002 | 62 | 44218 | 40737 | 0.681966 |
| 356.146 | 91 | 125852 | 131165 | 0.802927 | 612.883 | 61 | 257196 | 258846 | 0.947662 |
| 359.012 | 559 | 79745 | 104340 | 0.652046 | 631.145 | 537 | 192707 | 165557 | 0.423035 |
| 364.063 | 150 | 73935 | 96332 | 0.364811 | 641.995 | 554 | 61448 | 28854 | 0.024284 |
| 381.548 | 546 | 334135 | 310377 | 0.744735 | 698.228 | 452 | 44067 | 27290 | 0.050007 |
| 382.051 | 541 | 57544 | 51414 | 0.673151 | 762.086 | 546 | 105211 | 90957 | 0.506847 |
