# Supplementary material for: A biplot correlation range for group-wise metabolite selection in mass spectrometry
Source: BioData Min. 2019 Feb 4;12:4. doi: 10.1186/s13040-019-0191-2 (PMC6360680; doi:10.1186/s13040-019-0191-2)
Supplement: Supplementary file 8 — Table S7. The average number of filtered variables in each layer and the averaged P-values for the one-layer and noise-layer structures from the BCS method. (DOCX 58 kb) [file 13040_2019_191_MOESM8_ESM.docx]

**Additional file 8: Table S2. Three hundred ten features with variable importance projection (VIP) score greater than and equal to 1 were listed from mitochondria between wild and thioredoxin-2 transgenic mice**

| mz | time | VIP |
| --- | --- | --- |
| 132.1015 | 73.65223 | 0.978151 |
| 134.0443 | 167.7529 | 1.047099 |
| 135.0476 | 174.0033 | 1.002308 |
| 141.0136 | 536.5755 | 1.001129 |
| 145.1218 | 466.6495 | 1.079794 |
| 147.0297 | 151.8578 | 0.927515 |
| 147.0762 | 75.68975 | 0.901148 |
| 147.1131 | 62.04356 | 1.197225 |
| 148.0595 | 105.7336 | 0.897749 |
| 150.058 | 75.15394 | 0.945886 |
| 151.096 | 500.3845 | 0.786219 |
| 165.0749 | 144.0996 | 1.039624 |
| 166.0858 | 74.80001 | 0.934392 |
| 168.0686 | 75.63137 | 1.121054 |
| 182.0807 | 75.32251 | 0.972848 |
| 183.0855 | 192.6137 | 0.579117 |
| 183.0856 | 511.0823 | 1.279847 |
| 184.0635 | 78.91051 | 1.052449 |
| 184.0728 | 400.0393 | 1.446466 |
| 184.0889 | 170.0283 | 1.182211 |
| 189.1238 | 67.33045 | 1.035769 |
| 190.0704 | 390.4144 | 0.810312 |
| 196.1 | 75.7232 | 1.006829 |
| 196.956 | 246.5802 | 1.246714 |
| 198.0793 | 77.67281 | 1.057945 |
| 199.9871 | 29.88927 | 1.349991 |
| 200.1121 | 211.5618 | 0.864875 |
| 200.9717 | 447.4405 | 1.112864 |
| 203.1395 | 66.49265 | 0.996535 |
| 204.9577 | 60.60643 | 1.072193 |
| 205.0676 | 129.0093 | 1.533531 |
| 205.0966 | 75.86731 | 0.873148 |
| 205.9505 | 441.9723 | 0.889435 |
| 206.0713 | 75.54679 | 1.568237 |
| 209.1893 | 432.369 | 0.822789 |
| 210.1153 | 75.06376 | 0.961962 |
| mz | time | VIP |
| 211.1192 | 75.12643 | 1.073158 |
| 212.0579 | 167.7549 | 1.115791 |
| 213.0072 | 366.0603 | 0.87355 |
| 213.0611 | 161.8252 | 0.92658 |
| 214.5522 | 177.9822 | 1.2175 |
| 215.1395 | 72.98829 | 0.985926 |
| 215.5501 | 189.5918 | 1.255893 |
| 218.0102 | 406.0818 | 0.930639 |
| 219.1345 | 67.98789 | 1.077813 |
| 221.0414 | 113.1072 | 0.461963 |
| 223.1413 | 503.5974 | 0.620042 |
| 228.0722 | 76.53747 | 1.056194 |
| 229.1552 | 71.59469 | 1.001474 |
| 231.0256 | 432.0485 | 1.200744 |
| 231.1709 | 66.29633 | 0.976291 |
| 233.0462 | 382.8614 | 0.775078 |
| 233.1501 | 66.81584 | 0.957887 |
| 235.0089 | 514.3435 | 1.064906 |
| 237.124 | 66.44002 | 0.973508 |
| 239.0407 | 543.331 | 1.097856 |
| 239.1052 | 159.9128 | 0.716925 |
| 240.109 | 75.55425 | 0.549017 |
| 243.0254 | 528.0532 | 1.366325 |
| 244.0999 | 76.13112 | 1.069528 |
| 244.1379 | 164.8299 | 0.61402 |
| 245.1866 | 65.88723 | 0.978123 |
| 246.1453 | 69.86499 | 1.068886 |
| 246.1823 | 61.11636 | 0.995994 |
| 248.052 | 529.0163 | 1.620946 |
| 248.0521 | 290.135 | 0.94201 |
| 251.9554 | 560.8917 | 1.169775 |
| 252.9898 | 560.7308 | 1.017911 |
| 253.0073 | 433.9965 | 1.247646 |
| 253.1173 | 542.3661 | 0.470149 |
| 257.9216 | 533.6188 | 0.782873 |
| 258.1085 | 106.655 | 1.375651 |
| mz | time | VIP |
| 260.0517 | 518.67 | 1.503605 |
| 260.0951 | 76.81795 | 0.995766 |
| 260.161 | 67.77539 | 1.00387 |
| 260.1979 | 58.58754 | 1.033273 |
| 261.0955 | 121.1694 | 0.543205 |
| 261.1448 | 68.31742 | 0.976055 |
| 263.1398 | 69.71693 | 0.914204 |
| 265.1554 | 67.03606 | 0.950019 |
| 267.0812 | 187.0008 | 1.087518 |
| 267.0971 | 443.559 | 1.140656 |
| 267.9683 | 161.401 | 0.914043 |
| 268.1034 | 75.32406 | 0.893668 |
| 268.1378 | 168.436 | 0.925643 |
| 271.0127 | 560.6868 | 1.208232 |
| 272.9454 | 60.04362 | 1.014349 |
| 273.0917 | 246.3118 | 0.922006 |
| 274.0725 | 387.7884 | 0.645886 |
| 275.2567 | 490.2655 | 0.786756 |
| 275.9915 | 560.9402 | 1.327757 |
| 277.1018 | 342.3053 | 0.867047 |
| 278.0624 | 30.51477 | 1.040408 |
| 278.0624 | 425.7421 | 1.191786 |
| 278.1052 | 326.4604 | 1.251983 |
| 279.1708 | 66.84373 | 0.955619 |
| 279.6458 | 416.1871 | 1.458906 |
| 280.0918 | 74.13967 | 0.792712 |
| 281.0621 | 531.891 | 1.363792 |
| 282.002 | 246.019 | 0.705163 |
| 282.0767 | 171.6676 | 0.67211 |
| 282.2777 | 158.8482 | 0.948731 |
| 282.5775 | 139.0744 | 0.874895 |
| 283.0099 | 245.9632 | 0.751165 |
| 283.0817 | 76.79004 | 0.804554 |
| 284.0858 | 78.92102 | 0.623106 |
| 287.6321 | 416.641 | 0.995091 |
| 288.204 | 60.94228 | 1.004397 |
| 289.2724 | 432.9589 | 0.756595 |
| 290.0628 | 178.2621 | 0.572747 |
| mz | time | VIP |
| 290.1329 | 157.7238 | 1.345483 |
| 294.0105 | 550.551 | 0.895119 |
| 294.1456 | 68.20265 | 0.959582 |
| 294.6905 | 60.9647 | 1.001068 |
| 295.1661 | 67.42987 | 1.048114 |
| 297.1067 | 534.5319 | 0.487216 |
| 297.9869 | 560.9805 | 1.341199 |
| 299.0543 | 167.4588 | 1.149341 |
| 299.0607 | 470.8533 | 1.092109 |
| 299.0803 | 466.8342 | 0.672443 |
| 299.1261 | 77.51696 | 1.025921 |
| 300.2878 | 145.8032 | 1.163187 |
| 301.2912 | 158.9715 | 1.384029 |
| 303.9499 | 561.7922 | 1.275475 |
| 304.0227 | 563.0787 | 1.114225 |
| 305.0635 | 153.9191 | 0.854832 |
| 305.1516 | 205.8541 | 0.411773 |
| 306.0473 | 475.2071 | 1.186353 |
| 306.128 | 186.1063 | 0.731931 |
| 307.0508 | 459.9064 | 0.985474 |
| 307.0816 | 519.2771 | 0.472509 |
| 307.5838 | 518.6338 | 0.480218 |
| 307.5931 | 182.3767 | 0.969117 |
| 307.9806 | 168.25 | 0.991506 |
| 308.0771 | 478.9009 | 0.5793 |
| 308.0897 | 162.95 | 0.603188 |
| 308.5815 | 520.0362 | 0.639376 |
| 308.9322 | 560.4344 | 1.015958 |
| 309.0928 | 177.4012 | 0.665644 |
| 310.0855 | 180.5982 | 0.776615 |
| 310.112 | 552.9213 | 1.245296 |
| 311.1005 | 462.793 | 0.624151 |
| 312.0387 | 560.9986 | 1.579234 |
| 312.1035 | 445.7684 | 0.820682 |
| 313.911 | 560.2339 | 1.008694 |
| 314.0088 | 128.6256 | 0.957397 |
| 314.1066 | 169.1972 | 0.780563 |
| 315.0165 | 123.6048 | 1.005324 |
| mz | time | VIP |
| 315.1213 | 78.60069 | 1.526579 |
| 316.1222 | 167.4234 | 1.075433 |
| 319.246 | 471.6053 | 1.121843 |
| 319.9531 | 268.8037 | 1.022043 |
| 319.9533 | 511.939 | 1.021595 |
| 319.9533 | 27.26644 | 0.91766 |
| 320.1263 | 203.0627 | 0.782211 |
| 320.4548 | 24.21508 | 0.885436 |
| 321.0832 | 159.1914 | 0.826225 |
| 321.1091 | 154.073 | 0.855707 |
| 321.1102 | 446.9313 | 0.916324 |
| 322.105 | 175.0421 | 0.900156 |
| 323.0738 | 448.808 | 1.124683 |
| 323.1252 | 88.95138 | 0.902624 |
| 323.1431 | 546.1375 | 0.762735 |
| 324.0575 | 298.4779 | 0.702269 |
| 326.1063 | 554.9092 | 1.046913 |
| 326.9657 | 560.6337 | 1.276811 |
| 327.0528 | 63.64283 | 0.987149 |
| 327.1098 | 554.0039 | 1.079184 |
| 327.1573 | 77.72101 | 1.024665 |
| 328.0286 | 448.4879 | 1.122839 |
| 329.0695 | 155.5177 | 0.708911 |
| 332.0739 | 544.9516 | 1.093753 |
| 332.0817 | 512.4113 | 1.048683 |
| 332.1014 | 84.3917 | 0.77918 |
| 332.1255 | 89.88257 | 0.654854 |
| 332.5604 | 546.6419 | 1.190057 |
| 333.062 | 549.6978 | 1.187325 |
| 333.5628 | 555.0417 | 1.02381 |
| 337.0416 | 155.1313 | 0.847253 |
| 339.0574 | 167.8518 | 1.022407 |
| 339.1014 | 76.35996 | 0.787883 |
| 340.9332 | 59.914 | 1.095387 |
| 341.0898 | 168.1893 | 1.107953 |
| 341.1718 | 78.11587 | 0.923894 |
| 342.0681 | 33.87031 | 1.39579 |
| 342.1763 | 75.35455 | 0.933875 |
| mz | time | VIP |
| 343.9572 | 559.6601 | 1.400844 |
| 344.0997 | 94.51448 | 1.117669 |
| 344.9758 | 561.9822 | 1.187745 |
| 345.05 | 176.8486 | 1.546648 |
| 345.0754 | 143.6404 | 0.568262 |
| 345.1106 | 157.9606 | 0.820616 |
| 345.137 | 180.7159 | 0.995381 |
| 345.9809 | 559.3149 | 1.077745 |
| 345.9963 | 561.2087 | 1.104928 |
| 346.0395 | 358.0523 | 0.597685 |
| 346.089 | 519.7491 | 0.832974 |
| 348.0683 | 551.1465 | 0.868752 |
| 348.0683 | 242.4073 | 0.87681 |
| 348.2726 | 424.1134 | 1.242461 |
| 349.0717 | 552.1115 | 0.916884 |
| 349.9547 | 559.6266 | 1.245596 |
| 350.0721 | 553.6163 | 0.923354 |
| 350.2155 | 171.6445 | 1.016137 |
| 352.2367 | 511.7402 | 1.263605 |
| 352.9504 | 540.319 | 1.108213 |
| 353.0646 | 168.7176 | 1.16042 |
| 354.0219 | 154.3233 | 0.995452 |
| 354.6065 | 138.6289 | 1.13588 |
| 354.9367 | 562.1978 | 1.040316 |
| 355.0297 | 152.8898 | 0.980834 |
| 355.2611 | 175.9659 | 1.212067 |
| 356.1461 | 90.92037 | 1.050592 |
| 357.1291 | 105.6552 | 0.847194 |
| 357.2769 | 299.0924 | 0.938174 |
| 358.1149 | 95.49485 | 1.339755 |
| 358.1317 | 123.4414 | 0.873683 |
| 358.2804 | 221.1085 | 0.820194 |
| 359.0118 | 558.7018 | 1.449965 |
| 359.1276 | 94.56302 | 0.970571 |
| 361.9917 | 561.2506 | 1.201209 |
| 363.9526 | 558.7978 | 1.249729 |
| 364.0071 | 560.2636 | 1.172688 |
| 364.0628 | 150.2747 | 1.079534 |
| mz | time | VIP |
| 364.0631 | 510.7915 | 1.112527 |
| 365.8816 | 560.0163 | 0.735241 |
| 367.1691 | 561.9649 | 0.600634 |
| 367.9923 | 561.1002 | 1.231674 |
| 368.9937 | 557.9536 | 1.045384 |
| 371.5668 | 558.8644 | 0.900939 |
| 372.141 | 170.2167 | 0.726216 |
| 373.0622 | 189.0043 | 0.650505 |
| 373.1152 | 157.6584 | 0.744421 |
| 373.2712 | 170.1902 | 1.28015 |
| 373.6161 | 142.1933 | 0.851148 |
| 373.968 | 559.3036 | 1.124326 |
| 373.9707 | 104.265 | 0.72396 |
| 374.1275 | 531.297 | 0.930683 |
| 374.2169 | 53.79697 | 0.848267 |
| 374.2746 | 173.1097 | 1.284881 |
| 374.4673 | 53.2 | 0.735646 |
| 375.1168 | 155.4836 | 0.488692 |
| 376.054 | 556.8435 | 0.882526 |
| 377.0222 | 538.467 | 1.408994 |
| 377.0533 | 159.0011 | 0.956815 |
| 379.0819 | 469.9183 | 0.857872 |
| 380.0013 | 560.9323 | 1.113408 |
| 380.0928 | 181.7131 | 0.956633 |
| 381.1481 | 547.3898 | 0.773094 |
| 381.5479 | 546.2904 | 1.013586 |
| 382.0181 | 575.0234 | 0.88482 |
| 382.0507 | 541.3152 | 0.898888 |
| 384.0605 | 449.7255 | 1.493078 |
| 384.1125 | 497.7325 | 0.75142 |
| 385.1158 | 492.5004 | 0.566815 |
| 386.6112 | 143.2474 | 0.773322 |
| 387.0245 | 558.2773 | 0.901201 |
| 387.1448 | 161.279 | 0.843435 |
| 389.1735 | 353.3935 | 0.810407 |
| 389.2667 | 286.9712 | 1.18781 |
| 391.153 | 74.66929 | 1.01355 |
| 392.0219 | 122.3343 | 0.975026 |
| mz | time | VIP |
| 392.1809 | 334.6532 | 1.015921 |
| 393.615 | 86.72616 | 0.563037 |
| 394.2411 | 173.431 | 0.892659 |
| 394.2771 | 169.4934 | 0.772703 |
| 394.5969 | 135.1461 | 0.663537 |
| 395.0394 | 93.52575 | 0.960369 |
| 396.1378 | 568.036 | 0.801796 |
| 398.0665 | 553.4599 | 1.112371 |
| 398.1264 | 525.6265 | 0.672731 |
| 398.9988 | 535.9911 | 1.137947 |
| 399.0696 | 562.1451 | 1.00727 |
| 400.0198 | 558.2997 | 1.140347 |
| 400.219 | 67.85064 | 0.870514 |
| 401.2323 | 60.17115 | 0.966756 |
| 401.5662 | 55.92285 | 0.94737 |
| 401.9259 | 561.4569 | 1.16549 |
| 402.0709 | 275.5512 | 1.091222 |
| 404.5819 | 169.1887 | 1.141015 |
| 406.1439 | 553.0052 | 0.907327 |
| 406.9083 | 559.8415 | 0.685344 |
| 407.0084 | 164.3986 | 1.067875 |
| 407.2289 | 68.56893 | 0.88949 |
| 407.2767 | 209.3715 | 1.139147 |
| 408.0884 | 547.5679 | 0.885402 |
| 408.2908 | 373.3704 | 1.092341 |
| 408.9206 | 60.50079 | 1.039787 |
| 410.324 | 398.4772 | 1.091582 |
| 412.064 | 200.4703 | 1.097463 |
| 412.1935 | 168.3034 | 0.873174 |
| 412.5678 | 178.3851 | 1.126857 |
| 413.103 | 208.952 | 0.821546 |
| 413.1975 | 177.7906 | 0.8735 |
| 414.1371 | 96.77484 | 0.789429 |
| 414.9098 | 54.94017 | 0.671838 |
| 415.1181 | 220.6869 | 1.0373 |
| 415.1407 | 90.58083 | 0.978455 |
| 416.0583 | 179.8236 | 0.896778 |
| 416.1371 | 157.5987 | 0.997489 |
| mz | time | VIP |
| 416.8986 | 542.8786 | 0.664496 |
| 417.0703 | 157.3879 | 1.087545 |
| 418.0737 | 167.8958 | 0.804066 |
| 418.6941 | 169.8761 | 1.009563 |
| 419.2348 | 57.98231 | 1.054073 |
| 419.2623 | 28.35464 | 0.560537 |
| 419.4353 | 57.11638 | 0.967128 |
| 419.6362 | 56.03673 | 1.091939 |
| 422.0009 | 537.8117 | 1.033125 |
| 423.0874 | 548.9858 | 0.969078 |
| 424.0817 | 550.0095 | 0.939885 |
| 424.0998 | 561.1793 | 1.179495 |
| 424.9435 | 556.4864 | 1.207607 |
| 425.0865 | 544.7447 | 0.810279 |
| 425.8603 | 559.0903 | 1.078408 |
| 425.9389 | 560.1923 | 0.633799 |
| 426.0251 | 570.1384 | 1.032699 |
| 426.1661 | 457.7373 | 1.072668 |
| 426.3186 | 170.8441 | 1.373291 |
| 427.0915 | 167.3966 | 1.016765 |
| 427.1062 | 160.399 | 1.319271 |
| 427.1706 | 443.6893 | 0.772787 |
| 427.3219 | 172.0794 | 1.367668 |
| 428.096 | 169.3268 | 1.105304 |
| 428.0966 | 439.4032 | 1.136866 |
| 428.3246 | 172.5209 | 1.325379 |
| 428.6126 | 173.0933 | 0.938205 |
| 428.9568 | 531.7223 | 1.209611 |
| 429.095 | 147.7212 | 1.128233 |
| 430.0578 | 531.2714 | 0.849098 |
| 430.0914 | 166.1089 | 1.093635 |
| 430.5359 | 539.0064 | 1.116197 |
| 431.0381 | 538.0472 | 0.819048 |
| 431.0947 | 166.8788 | 1.08324 |
| 431.2739 | 166.9122 | 1.385919 |
| 432.0347 | 150.0727 | 0.855723 |
| 432.0887 | 174.9441 | 1.069462 |
| 432.1685 | 104.7444 | 1.318512 |
| mz | time | VIP |
| 432.2769 | 171.157 | 1.353395 |
| 433.0432 | 151.2862 | 0.810533 |
| 433.1747 | 83.05473 | 0.919915 |
| 434.0593 | 449.0019 | 1.159541 |
| 434.0594 | 34.04017 | 0.465604 |
| 435.0629 | 536.6379 | 0.916975 |
| 436.0641 | 557.8646 | 1.126063 |
| 436.5987 | 176.5719 | 0.917439 |
| 437.5655 | 56.0216 | 0.952474 |
| 438.1215 | 91.91787 | 0.998894 |
| 438.2282 | 72.98152 | 0.974517 |
| 439.0984 | 29.9004 | 1.018593 |
| 439.0987 | 269.8603 | 0.788948 |
| 439.099 | 534.9796 | 1.021187 |
| 439.595 | 545.4959 | 0.69113 |
| 440.1074 | 505.5629 | 0.804105 |
| 442.758 | 59.69568 | 0.875436 |
| 443.1932 | 450.0516 | 0.804885 |
| 443.559 | 54.20876 | 0.76405 |
| 443.8937 | 53.84897 | 0.753167 |
| 443.9721 | 558.3933 | 1.203034 |
| 444.1603 | 241.3135 | 1.058945 |
| 444.2091 | 65.38903 | 0.815348 |
| 445.1432 | 110.9465 | 1.220956 |
| 445.6452 | 126.5032 | 1.052659 |
| 446.044 | 554.6802 | 0.956605 |
| 446.1773 | 169.6328 | 0.75006 |
| 446.7233 | 58.75254 | 0.882562 |
| 447.0644 | 207.3693 | 1.103585 |
| 447.2471 | 116.5278 | 1.126232 |
| 447.498 | 55.82923 | 0.802687 |
| 448.1482 | 453.5315 | 0.475722 |
| 448.1695 | 141.5508 | 1.372193 |
| 449.0099 | 53.43253 | 0.954589 |
| 449.1812 | 76.20637 | 0.763142 |
| 450.0768 | 173.9773 | 1.116758 |
| 450.9259 | 543.062 | 0.977696 |
| 450.9391 | 533.6467 | 1.182482 |
| mz | time | VIP |
| 451.1059 | 185.0285 | 0.817417 |
| 453.1324 | 166.0949 | 0.433385 |
| 453.6347 | 167.2216 | 0.431375 |
| 454.1359 | 168.769 | 0.695766 |
| 455.4174 | 56.22491 | 0.874569 |
| 456.1686 | 86.81894 | 0.797214 |
| 457.0823 | 155.9057 | 0.855812 |
| 457.1093 | 529.6274 | 1.03862 |
| 457.4957 | 542.1898 | 1.121794 |
| 460.0691 | 185.0008 | 0.64818 |
| 460.1203 | 170.152 | 1.227863 |
| 460.2008 | 78.89286 | 0.901263 |
| 461.1191 | 164.1941 | 0.569391 |
| 461.2053 | 74.2271 | 0.986625 |
| 461.6203 | 161.1347 | 0.766538 |
| 462.1211 | 160.1136 | 1.029582 |
| 462.1796 | 80.82107 | 0.895162 |
| 463.0274 | 63.79725 | 1.045038 |
| 463.8566 | 559.1168 | 0.454971 |
| 464.0779 | 565.7334 | 1.025145 |
| 465.1448 | 82.58292 | 0.487349 |
| 465.7436 | 55.27093 | 0.931483 |
| 465.9945 | 54.8093 | 0.986958 |
| 466.049 | 220.2915 | 1.231238 |
| 466.2445 | 56.20078 | 0.875376 |
| 466.9038 | 543.8586 | 1.24737 |
| 467.1803 | 81.15511 | 1.491481 |
| 467.5312 | 554.6366 | 0.730174 |
| 468.0261 | 556.6194 | 0.816175 |
| 468.5272 | 54.34164 | 0.779255 |
| 468.7776 | 55.42111 | 0.662453 |
| 469.121 | 162.2362 | 1.224407 |
| 470.1066 | 548.9928 | 1.125214 |
| 470.1582 | 76.53608 | 1.03866 |
| 470.3437 | 172.6319 | 0.814786 |
| 470.9261 | 537.7442 | 1.102214 |
| 471.1641 | 76.00247 | 1.056539 |
| 472.0468 | 157.4571 | 0.892958 |
| mz | time | VIP |
| 472.0606 | 222.6865 | 1.05081 |
| 472.0845 | 27.92448 | 0.86448 |
| 472.1548 | 309.4552 | 1.055797 |
| 473.0552 | 158.9899 | 0.910805 |
| 474.2145 | 133.8377 | 0.768948 |
| 475.2196 | 73.90392 | 0.94591 |
| 475.5174 | 545.4809 | 0.697101 |
| 476.158 | 167.2201 | 0.643645 |
| 476.6475 | 210.5547 | 1.027355 |
| 476.9085 | 60.61834 | 0.980573 |
| 477.0595 | 174.8431 | 0.796053 |
| 477.1611 | 167.7587 | 0.640697 |
| 477.1757 | 75.76647 | 0.916757 |
| 477.9098 | 60.54961 | 1.219328 |
| 478.1388 | 521.0698 | 0.557095 |
| 478.1622 | 167.4953 | 0.638202 |
| 478.29 | 562.6249 | 1.212268 |
| 478.6404 | 519.3638 | 0.881365 |
| 478.9379 | 55.228 | 1.021732 |
| 479.0194 | 55.32395 | 0.872306 |
| 479.1074 | 491.5332 | 0.978141 |
| 479.1653 | 166.9092 | 0.858888 |
| 479.2463 | 404.7026 | 0.833885 |
| 479.2705 | 54.50915 | 0.941036 |
| 479.293 | 556.3682 | 1.196443 |
| 479.5208 | 55.46394 | 0.757103 |
| 479.524 | 537.7165 | 1.415807 |
| 480.162 | 209.297 | 1.011 |
| 481.0067 | 196.4219 | 1.104376 |
| 481.1654 | 196.4797 | 0.743515 |
| 482.0151 | 192.6621 | 1.225688 |
| 482.8763 | 542.2834 | 1.233899 |
| 483.0996 | 214.1453 | 0.900923 |
| 483.5037 | 53.55613 | 1.125185 |
| 483.7533 | 55.5191 | 0.991771 |
| 484.1144 | 518.8748 | 1.057538 |
| 484.1184 | 172.9444 | 1.50587 |
| 485.2503 | 55.049 | 0.388472 |
| mz | time | VIP |
| 485.2921 | 57.68283 | 0.88301 |
| 485.5008 | 53.37514 | 1.177699 |
| 485.793 | 55.63336 | 0.727945 |
| 486.1343 | 75.70222 | 1.036444 |
| 487.3047 | 167.3834 | 1.296734 |
| 487.906 | 60.97864 | 0.847811 |
| 488.3087 | 168.137 | 1.308964 |
| 489.1633 | 79.39682 | 1.145726 |
| 489.1921 | 74.71275 | 0.840286 |
| 489.2275 | 72.12347 | 0.859682 |
| 489.3126 | 206.5901 | 0.920061 |
| 490.115 | 199.4835 | 1.111709 |
| 490.1726 | 142.4379 | 0.719785 |
| 491.517 | 54.31797 | 1.006348 |
| 491.7673 | 54.67106 | 1.016157 |
| 492.018 | 59.22027 | 1.015113 |
| 492.1735 | 78.2448 | 0.979018 |
| 492.2696 | 56.35218 | 1.025685 |
| 494.3207 | 354.3002 | 0.860032 |
| 495.0167 | 29.03132 | 1.145066 |
| 496.9735 | 555.7602 | 0.63076 |
| 497.1299 | 567.0271 | 1.31253 |
| 497.3391 | 544.147 | 1.056715 |
| 498.0638 | 202.5469 | 0.65396 |
| 498.2862 | 54.57407 | 0.745783 |
| 498.6204 | 54.68939 | 0.727452 |
| 499.0721 | 198.1569 | 0.675639 |
| 499.2877 | 55.59588 | 1.335935 |
| 500.2736 | 463.9858 | 0.855492 |
| 501.2775 | 445.7566 | 1.110173 |
| 502.2865 | 66.52172 | 0.961178 |
| 502.2903 | 566.0404 | 1.130957 |
| 503.2923 | 564.1466 | 1.106785 |
| 505.0457 | 548.7021 | 0.868334 |
| 505.9804 | 540.694 | 1.047804 |
| 508.2013 | 82.55383 | 1.059044 |
| 509.2085 | 103.4058 | 0.927893 |
| 509.2686 | 53.86116 | 1.200009 |
| mz | time | VIP |
| 509.2866 | 170.9129 | 1.421705 |
| 509.3571 | 155.7089 | 0.903408 |
| 509.5173 | 53.1815 | 0.574242 |
| 509.8091 | 459.4785 | 1.072352 |
| 510.1276 | 217.3377 | 0.625011 |
| 511.056 | 158.5855 | 1.197109 |
| 512.0607 | 160.5577 | 1.169183 |
| 512.1351 | 554.8489 | 0.879444 |
| 512.2923 | 53.09611 | 0.872213 |
| 514.3349 | 69.19073 | 0.776497 |
| 516.2956 | 541.2124 | 0.767951 |
| 516.296 | 275.1564 | 0.651001 |
| 516.296 | 32.57601 | 0.929001 |
| 516.6907 | 86.72427 | 0.718271 |
| 516.9467 | 55.00273 | 1.047854 |
| 517.3023 | 552.0577 | 0.514657 |
| 518.3214 | 308.159 | 1.26791 |
| 519.9369 | 55.14214 | 0.906625 |
| 520.272 | 54.41914 | 0.938602 |
| 520.3364 | 478.5894 | 1.416925 |
| 521.3388 | 479.0802 | 1.385363 |
| 521.8992 | 61.61518 | 0.935173 |
| 522.1987 | 206.4696 | 0.504286 |
| 522.3444 | 471.919 | 1.280204 |
| 523.5406 | 56.56869 | 1.000206 |
| 523.791 | 59.57447 | 0.99111 |
| 524.0427 | 56.96518 | 1.010826 |
| 524.0541 | 562.4178 | 1.447174 |
| 524.2935 | 56.59107 | 0.994624 |
| 524.3176 | 185.4951 | 1.122958 |
| 524.5448 | 58.09534 | 1.302464 |
| 524.953 | 558.1055 | 1.061256 |
| 526.9605 | 55.05462 | 0.887453 |
| 527.1547 | 177.3432 | 0.679977 |
| 527.637 | 169.9876 | 0.783105 |
| 528.2384 | 56.28204 | 0.852145 |
| 528.572 | 52.35291 | 1.215563 |
| 529.0683 | 188.1331 | 0.974308 |
| mz | time | VIP |
| 529.887 | 61.21834 | 0.961276 |
| 530.0759 | 178.2144 | 1.075777 |
| 531.0147 | 65.09453 | 0.995206 |
| 531.2784 | 56.46196 | 0.779247 |
| 532.1833 | 367.4595 | 1.060062 |
| 533.1866 | 326.019 | 0.924239 |
| 533.3211 | 449.5855 | 1.02474 |
| 533.3215 | 31.37454 | 1.38217 |
| 534.6383 | 55.52083 | 0.712985 |
| 534.8955 | 56.49207 | 0.907391 |
| 534.9728 | 55.99956 | 0.728039 |
| 536.9502 | 531.8212 | 0.707107 |
| 537.5375 | 53.88252 | 0.757261 |
| 537.7892 | 53.70954 | 1.333583 |
| 537.8035 | 553.7195 | 0.960615 |
| 542.0649 | 556.2279 | 1.012624 |
| 542.3008 | 53.56462 | 1.093688 |
| 542.322 | 316.3696 | 1.208995 |
| 542.5019 | 53.07709 | 0.936824 |
| 542.7025 | 52.82683 | 0.921818 |
| 543.3255 | 298.8606 | 1.258638 |
| 544.171 | 167.4816 | 0.665933 |
| 544.3306 | 133.3907 | 0.863431 |
| 544.3365 | 486.8888 | 1.316392 |
| 544.7389 | 558.3402 | 1.312098 |
| 544.8968 | 60.90738 | 0.98583 |
| 545.3402 | 483.7142 | 1.3119 |
| 545.8972 | 60.15415 | 0.933794 |
| 546.0991 | 55.70421 | 0.412414 |
| 546.299 | 56.11982 | 0.686252 |
| 546.3466 | 500.0473 | 1.155004 |
| 546.5007 | 56.29919 | 0.459007 |
| 547.1809 | 223.4784 | 0.566196 |
| 547.3556 | 455.2274 | 1.1794 |
| 548.1912 | 72.98139 | 0.536788 |
| 550.1137 | 283.9653 | 1.035907 |
| 552.157 | 170.0646 | 0.547162 |
| 555.893 | 61.08561 | 0.973142 |
| mz | time | VIP |
| 556.1235 | 319.2008 | 1.520893 |
| 556.6644 | 169.7097 | 1.094509 |
| 557.1658 | 173.8231 | 1.11864 |
| 557.6673 | 176.8994 | 1.132711 |
| 557.7991 | 54.5752 | 0.919778 |
| 562.1505 | 431.6496 | 1.125582 |
| 563.8799 | 61.32664 | 0.897883 |
| 564.8785 | 543.3047 | 1.217067 |
| 564.9754 | 54.0045 | 1.031695 |
| 565.3083 | 54.67503 | 0.95887 |
| 566.2359 | 173.0308 | 1.238517 |
| 566.3079 | 60.24079 | 1.848603 |
| 566.3171 | 434.0935 | 1.317721 |
| 568.3349 | 504.5726 | 1.135587 |
| 569.1629 | 203.7388 | 0.870353 |
| 569.3381 | 500.9637 | 1.121588 |
| 570.3454 | 502.1359 | 1.171639 |
| 571.2896 | 59.20597 | 0.86158 |
| 571.3434 | 173.0133 | 1.3372 |
| 571.8062 | 54.03674 | 0.784968 |
| 571.9744 | 53.6691 | 0.948222 |
| 572.1415 | 53.22718 | 1.03175 |
| 573.1389 | 310.3161 | 1.278604 |
| 575.3022 | 58.07076 | 0.860688 |
| 575.6378 | 179.0326 | 1.089427 |
| 578.1093 | 203.9869 | 0.702929 |
| 579.1164 | 217.2276 | 0.733031 |
| 580.8505 | 535.8046 | 1.194475 |
| 581.2211 | 137.6052 | 1.347396 |
| 583.6235 | 183.2257 | 1.012348 |
| 585.1527 | 81.20553 | 1.052576 |
| 589.1362 | 215.082 | 0.819044 |
| 589.8876 | 61.2347 | 0.873477 |
| 590.3172 | 458.1995 | 1.302412 |
| 591.183 | 221.2193 | 1.4147 |
| 591.3683 | 173.6825 | 1.369373 |
| 595.1197 | 285.4805 | 0.916587 |
| 595.659 | 55.65238 | 1.065656 |
| mz | time | VIP |
| 595.9925 | 55.19749 | 0.922561 |
| 597.1513 | 435.6085 | 0.846662 |
| 599.0024 | 61.95734 | 0.826587 |
| 599.1216 | 240.0166 | 0.85972 |
| 599.6683 | 174.207 | 0.779838 |
| 600.9225 | 531.0953 | 1.167315 |
| 601.3442 | 58.40928 | 0.939956 |
| 603.2018 | 74.84711 | 0.627018 |
| 604.2052 | 75.16622 | 0.585839 |
| 609.6542 | 55.06579 | 0.936164 |
| 612.3238 | 54.82714 | 1.006055 |
| 612.6566 | 54.61283 | 0.984611 |
| 612.8828 | 61.36407 | 1.021227 |
| 612.991 | 55.81419 | 1.071536 |
| 613.1554 | 520.5986 | 0.752773 |
| 614.1593 | 523.3565 | 0.733336 |
| 615.0932 | 210.6578 | 1.349103 |
| 615.1481 | 517.0179 | 0.817374 |
| 617.3171 | 54.00794 | 1.008817 |
| 619.2155 | 301.4555 | 1.414417 |
| 622.2053 | 76.99742 | 0.564649 |
| 624.1884 | 178.0296 | 0.878329 |
| 624.69 | 183.8986 | 0.996276 |
| 628.3789 | 67.89996 | 0.972902 |
| 628.8577 | 61.94203 | 1.279066 |
| 631.1452 | 537.3693 | 0.926587 |
| 631.8669 | 62.21223 | 0.918315 |
| 632.1467 | 532.2935 | 0.48947 |
| 632.174 | 175.9284 | 0.959998 |
| 632.6763 | 182.299 | 0.641205 |
| 633.158 | 219.2025 | 1.017071 |
| 639.8529 | 59.05527 | 0.887096 |
| 641.1143 | 52.27496 | 0.58494 |
| 641.3176 | 52.28475 | 1.033513 |
| 641.5166 | 52.18788 | 1.144082 |
| 641.7163 | 52.34721 | 0.739641 |
| 641.9951 | 553.8619 | 1.315235 |
| 645.361 | 57.48555 | 0.660387 |
| mz | time | VIP |
| 645.6124 | 55.29982 | 0.680329 |
| 645.8582 | 54.28301 | 1.005844 |
| 648.1608 | 392.1406 | 1.627524 |
| 655.0212 | 54.88607 | 0.960315 |
| 655.3547 | 54.50234 | 0.965494 |
| 655.6899 | 55.94331 | 1.031255 |
| 656.0238 | 56.88921 | 0.888086 |
| 662.8533 | 540.0385 | 1.222224 |
| 663.1926 | 479.5468 | 1.098993 |
| 664.1106 | 549.6831 | 1.131462 |
| 665.1158 | 552.6489 | 1.071668 |
| 665.862 | 61.25567 | 0.942558 |
| 666.1143 | 563.0582 | 1.219326 |
| 666.3717 | 53.32533 | 1.023032 |
| 666.519 | 53.26317 | 0.547673 |
| 666.665 | 52.48844 | 1.071657 |
| 667.2226 | 209.1683 | 0.577551 |
| 680.8716 | 61.38323 | 1.115655 |
| 681.2027 | 462.4957 | 1.054949 |
| 681.2391 | 54.59813 | 0.992849 |
| 681.3631 | 54.7606 | 0.910438 |
| 681.4923 | 53.36383 | 0.610737 |
| 690.1806 | 558.9794 | 0.753858 |
| 696.8464 | 62.50987 | 0.605588 |
| 697.7184 | 57.2081 | 0.974581 |
| 698.0539 | 56.30271 | 1.154724 |
| 698.2279 | 452.0448 | 1.094976 |
| 698.385 | 57.31658 | 0.854704 |
| 699.8548 | 61.77594 | 1.078055 |
| 735.0239 | 530.8787 | 0.981156 |
| 748.8601 | 61.3209 | 0.76871 |
| 762.0858 | 546.1165 | 0.859361 |
| 778.2895 | 80.01304 | 0.719364 |
| 779.3993 | 58.72123 | 0.81333 |
| 779.9056 | 56.01607 | 0.772542 |
